# Spatial distribution and ecological risk of potentially toxic elements in peri-urban soils of a historically industrialised area

**DOI:** 10.1007/s10661-025-14389-5

**Published:** 2025-07-24

**Authors:** Fabrizio Monaci, Davide Baroni

**Affiliations:** 1 Department of Life Sciences, University of Siena, Via Mattioli 4, Siena, 53100 Italy; 2 National Biodiversity Future Center (NBFC), Palermo, 90133 Italy; 3 Department of Physical, Earth and Environmental Sciences, University of Siena, Via Mattioli 4, Siena, 53100 Italy

**Keywords:** PTEs, Arsenic, Zinc, Copper, Topsoil, Pyrite roasting, Peri-urban area, Ecological risk

## Abstract

The spatial distribution and ecological risk of potentially toxic elements (PTEs) were investigated in the topsoil of a peri-urban area surrounding a historical industrial complex in the Scarlino Plain in southwestern Tuscany, Italy. Superficial (0–5 cm) soil samples from 44 sites within a 1.5-km radius of the industrial district were analysed for As, Cd, Co, Cr, Cu, Hg, Mn, Ni, Pb, Sb, Sn, Tl, V and Zn concentrations after microwave-assisted acid digestion with HNO_3_ and H_2_O_2_. The results revealed significant contamination exceeding regulatory thresholds, predominantly caused by historical pyrite roasting and natural geochemical anomalies. The enrichment of As, Mn, Cu and Zn compared to European baselines was widespread, with As and Zn levels exceeding the Italian screening values in several samples. Principal component analysis identified distinct contamination patterns linked to land use, with the first component (PC1, 49.2% of the variance) associated with pyrite-derived PTEs (As, Cu, Cd, Pb and Zn), whereas PC2 represented geogenic inputs. Despite their combined contribution to soil contamination, this analysis effectively differentiated industrial signatures from those of natural sources. Ecological risk assessment indicated that As and Cd are major concerns in agricultural soils, where anthropogenic and natural sources overlap. These findings underscore the importance of distinguishing between natural and anthropogenic contributions to inform realistic mitigation targets and protect environmental and human health in historically impacted peri-urban areas.

**Supplementary Information:**

The online version contains supplementary material available at 10.1007/s10661-025-14389-5.

## Introduction

Soil is a crucial ecosystem for the biosphere and humankind, regulating life-supporting biogeochemical cycles and providing essential ecological services, such as biotic diversity, water quality, and global food security (Amundson et al., 2015; Ponge, 2015). Conservation of the ecological functionality of soils has recently become a global concern, as they are increasingly endangered by environmental changes and human activities (Foley et al., 2005; Lehmann et al., 2020). Among the latter, unsustainable farming practices, inadequately regulated industrial or mining activities and poor waste management are the most prevalent causes of long-term land degradation that harm soil fertility, composition and structure (Mai et al., 2025).

Chemical pollution is widely recognised as a significant threat to soil resources because it contributes to the degradation of diminishing fertile soils and has indirect negative consequences for agri-food systems and water safety (UNEP, 2021). Because of its conservative nature, soil is the ultimate repository of toxic chemicals in terrestrial environments and, at the same time, an important medium for human exposure (Bayabil et al., 2022; Picó et al., 2019). In the past, the distinctive capacity of soil compartments to retain heavy metals and other pollutants recalcitrant to degradation has been largely overlooked, leading to the creation of thousands of contaminated sites (i.e. brownsites) in Europe and the USA (Hou et al., 2023).

Soil contamination is most frequently associated with high concentrations of heavy metals and other potentially toxic elements (PTEs), mineral oils and persistent organic chemicals, such as chlorinated hydrocarbons (EEA, 2023; Pérez & Rodríguez, 2018). Among soil pollutants, PTEs such as arsenic (As), cadmium (Cd) and lead (Pb) are of special concern because their toxic, everlasting and often bioaccumulative nature is the origin of both historical environmental problems and pressing contemporary human health issues. A significant challenge that frequently hinders the accurate ecotoxicological assessment of PTEs in soil is distinguishing their sources. Elevated concentrations of these elements can result from both human activity and naturally occurring geological anomalies (Cai et al., 2024), complicating the process of determining the true risk associated with the presence of PTEs in soils. Recent advances have shown that the baseline concentrations of PTEs in both soil and stream sediments can be strongly modulated by lithology as well as by long-term industrial and urban inputs. In mineralised terranes, primary geogenic enrichment elevates the background levels of chalcophile elements, such As, Cu, Cd and Zn, in soils, as illustrated for pre-mining porphyry-copper districts in central Iran (Modabberi et al., 2023). Similar lithogenic signatures appear in stream sediments draining ultramafic and volcanic sequences, where elemental assemblages directly reflect their source rocks (Ambrosino et al., 2024). Superimposed on this natural signal, peri-urban and industrial catchments often display discrete patterns of anthropogenic enrichment. Diffuse atmospheric fallout, road dust and legacy mining wastes enhance the relative abundance of Pb, Zn and Cu in soils (Tepanosyan et al., 2020), with comparable anthropogenic fingerprints observed in river-borne sediments downstream of large metropolitan areas (Ambrosino et al., 2023). Therefore, disentangling these overlapping sources is critical for a realistic ecotoxicological appraisal of PTEs in alluvial plain environments, particularly within heavily industrialised lowland basins.

Over the last three decades, a toolbox of complementary approaches has been developed worldwide for the quantification and interpretation of PTEs in soils. Field sampling now ranges from continental fixed‑grid designs (Salminen et al., 2005) to GIS-constrained–random schemes that optimise spatial representativeness (Vizard et al., 2006), while rapid site screening is increasingly performed with portable‑XRF devices that reproduce ICP‑MS data for Pb, Zn and As (Ravansari et al., 2020). In the laboratory, aqua-regia or mixed-acid microwave digestions (USEPA, 1996) followed by ICP-MS/OES remain the standard, and when chemical speciation is critical, sequential extractions such as the four-step BCR protocol (Rauret et al., 1999) or the classic Tessier scheme (Tessier et al., 1979) are routinely adopted.

To convert raw elemental data into environmentally relevant metrics, researchers couple traditional contamination indices, the enrichment factor, geo‑accumulation index and Nemerow index (Hoshyari et al., 2023), with ecological or human health frameworks such as Håkanson’s Potential Ecological Risk Index (Håkanson, 1980) and the US‑EPA HHRA model (USEPA, 2011). Finally, multivariate source-apportionment is performed using principal component analysis (PCA) on centred log-ratio (clr)-transformed data with CoDaPack (Comas-Cufí & Thió-Henestrosa, (2011)), whereas positive matrix factorization (PMF) is conducted separately with dedicated statistical tools. These approaches are commonly combined with geostatistical interpolation, ordinary and indicator kriging and geographically weighted regression (GWR) to visualise spatial patterns across contrasting settings, including steel-manufacturing districts in Central Europe (Agyeman et al., 2021), intensively cultivated peri-urban farmland in eastern China (Ren et al., 2022) and farmland adjoining electroplating and non-ferrous-metal smelting parks in the Yangtze River Delta (Shao et al., 2024).

More recently, researchers and national and international agencies aiming at environmental protection have shifted their focus beyond contaminated sites with a recognisable contamination history to include peri-urban areas and spaces at the interface of urbanised and rural regions, where complex land-use patterns and overlapping risks present unique challenges (Hu et al., 2023). Characterised by multifunctional land use (industrial, agricultural and residential), peri-urban areas face significant human and ecological risks due to the proximity of contamination sources (e.g. polluted soils) and chemical exposure targets (agriculture and residents) which are often overlooked. These soils frequently contain complex mixtures of PTEs and other persistent pollutants from multiple diffuse or point sources that accumulate over time, making environmental risks particularly difficult to assess (Golia et al., 2024; Kibblewhite, 2018; Wu et al., 2023). Although residents and decision-makers are generally aware of the potential for contamination, they often underestimate the implications of living and farming near these sites (Radziszewska-Zielina et al., 2022; Zwirowicz-Rutkowska et al., 2023). With peri-urban spaces expected to expand further due to rapid urbanisation, especially in the Global South (Rajendran et al., 2024), significant gaps persist regarding how these transitional landscapes influence the spatial distribution, source differentiation and ecological risk of PTEs.

This study addresses these gaps by systematically analysing the distribution and sources of PTEs in the Scarlino Plain, a peri-urban area in Tuscany, Italy. The investigation combines a GIS-constrained random sampling design that secures spatial representativeness inside a 1.5-km impact buffer with ICP-MS determinations of element concentrations. The area is characterised by complex land-use patterns shaped by decades of socioeconomic growth, encompassing commercial and industrial sites, agricultural fields, brownfields awaiting remediation, highway infrastructure and diverse residential developments. Similar to many post-industrial European regions undergoing urban renewal, the Scarlino Plain faces significant challenges, particularly land contamination and associated environmental risks. To address these issues, an environmental surveillance program was initiated in 2013 as part of an EU Environmental Impact Assessment (EIA) permitting procedure. Within this framework, we analysed the spatial distribution of PTEs in the topsoil across the Scarlino District, aiming to map their concentrations and identify their sources and distribution mechanisms. Our methodological approach incorporated several innovative features: (*i*) treatment of elemental concentrations as compositional data via centred log-ratio (clr) transformation to overcome the closure problem inherent in geochemical datasets; (*ii*) kriging of principal component scores derived from the clr-transformed data to produce spatially explicit maps of the main contamination sources and (*iii*) calculation of ecological risk indices using locally determined geochemical background values rather than generic thresholds, enabling more accurate risk assessment in an area with complex natural geochemical anomalies. Our primary objective was to conduct a preliminary risk assessment to guide remediation strategies and public interventions to mitigate the impact of soil contamination on the local ecosystem. Beyond addressing a key knowledge gap, the present work was designed to offer a transferable framework for source-apportioned risk assessment in peri-urban soils, thereby providing decision-makers with a solid evidence base for prioritising remediation under the EU Soil Strategy for 2030 and the UN Sustainable Development Goals. In doing so, it intends to highlight how legacy industry landscapes, such as the Scarlino Plain, can be reframed from potential liabilities into model laboratories for sustainable land management policies.

## Materials and methods

### Study area

**Fig. 1:**
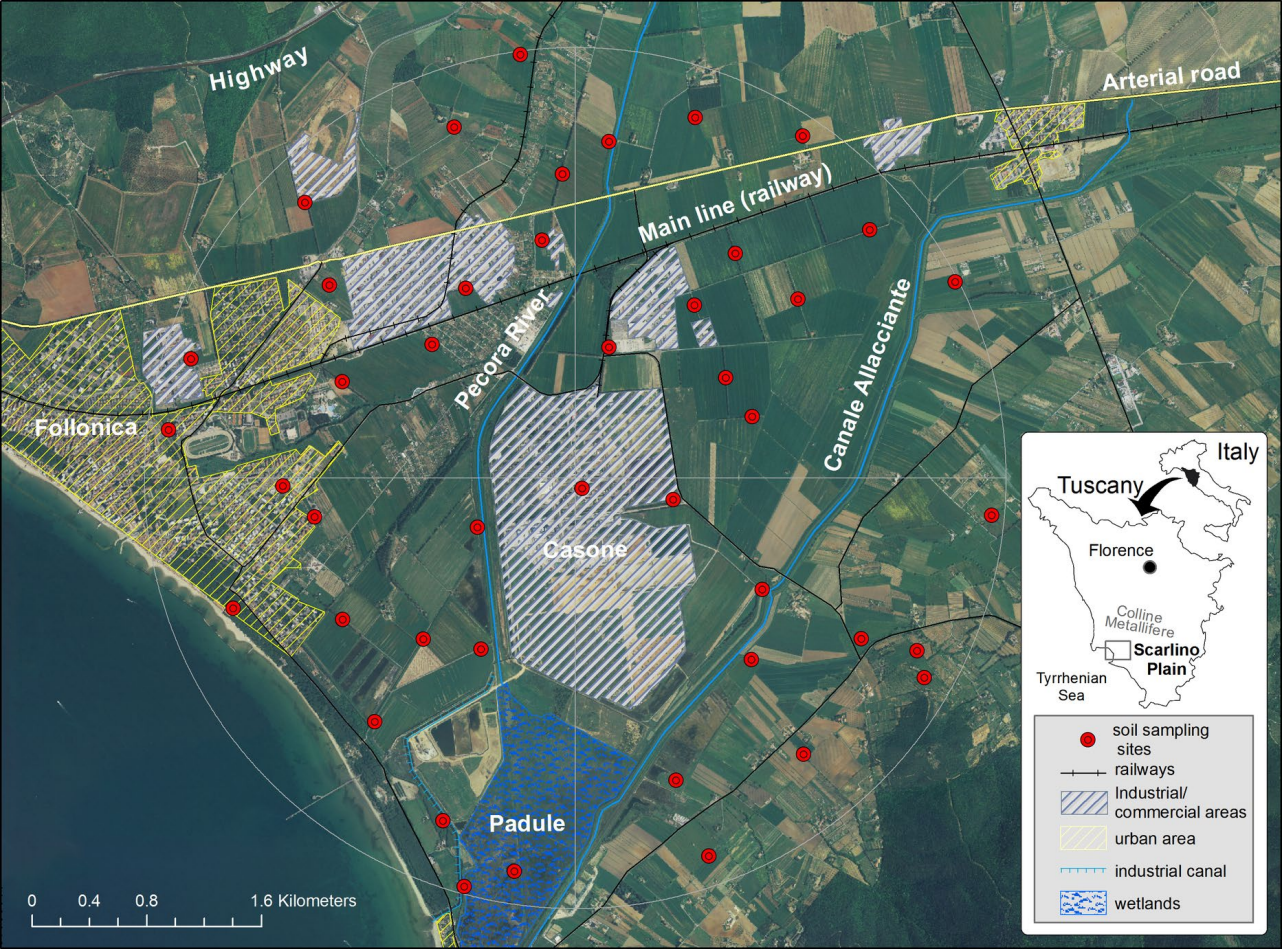
Location of the study area in the Scarlino Plain (southwestern Tuscany, Italy), with the main land use types (CORINE Land Cover, 2018) and distribution of soil sampling sites (*n* = 44)

The study area is situated on the Scarlino Plain, a flat coastal region in southwestern Tuscany, Central Italy, covering approximately 60 km^2^ (Fig. 1). The Plain faces the Gulf of Follonica and is enclosed in a basin delimited by hilly terrain reaching up to 500 m a.s.l. This basin is hydrologically connected to the Colline Metallifere region to the east-southeast via the Pecora River drainage basin, which represents the terminal sedimentary sector. The Pecora River originates in the mineralised area of the Colline Metallifere (northeast of the Scarlino Plain; Fig. 1, inset), which hosts several polymetallic ore bodies (e.g. the Massa Marittima district) exploited since the Villanovan period (ca. 1000 BC), with mining activity continuing through the Etruscan and Roman eras, intermittently during the medieval and Renaissance periods and on an industrial scale for sulphide roasting during the nineteenth to twentieth centuries (Rossato et al., 2011). Originally, the Scarlino Plain was occupied by a vast wetland that was gradually filled in 1830. Currently, the residual freshwater basin of this wetland, known as Padule (Fig. 1), is fed by the Pecora River before flowing into the Gulf of Follonica. To support agricultural and industrial development, the hydraulic network was enhanced by constructing the Canale Allacciante at the beginning of the twenty-first century. This canal drains water from creek systems in the northern Pecora Valley and serves as a critical component of the hydrological infrastructure of the plain. Together, the Pecora River and Canale Allacciante define the Casone area, which is historically the centre of a major industrial activity.

The area is characterised by a Mediterranean climate, with an average annual temperature of 15.9 °C and an annual average precipitation of 655 mm year^−1^, peaking in autumn and reaching its lowest levels in summer. The population is mainly concentrated in two urban areas: Follonica (21,800 inhabitants; NW coastal area; Fig. 1) and Scarlino Scalo (1500 inhabitants; inner valley, east). Sparse rural settlements are primarily involved in high-quality agricultural and tourism activities in these areas. From 1962 to 1994, the Casone area was dominated by a manufacturing enterprise that produced sulfuric acid and iron pellets from roasting pyrite sourced from deposits in the Colline Metallifere district. This process, which generated acid drainage, ash and pyrite tailings, significantly impacted the surrounding environment. Pyrite residues remain an environmental concern, prompting remediation efforts, groundwater containment measures and the recent approval of additional remediation projects to address these issues. Diffuse soil pollution across the Scarlino Plain is linked to the historical use of pyrite ash as a material for fills, embankments and roadbeds, a practice that was common among companies and individuals, as well as to the atmospheric fallout of pollutants emitted during smelting operations (ARPAT, 2015). By the mid-2010s, industrial activities at the Casone Complex had shifted predominantly to the basic chemical sector. Key operations include sulphuric acid production from petroleum, titanium dioxide production from ilmenite (treated with concentrated sulfuric acid produced on-site) and waste incineration. The latter began in the late 1990 s and involved the conversion of former pyrite roasting furnaces into an energy production facility using non-hazardous solid waste and biomass.

### Geological setting

The Scarlino Plain forms the seaward terminus of the Pecora River catchment, an ~ 240 km^2^ basin draining the western Colline Metallifere (Fig. 1). It occupies a NNW-SSE graben, where up to 200 m of Neogene–Quaternary sediments rest on a polydeformed Mesozoic–Palaeozoic basement (Rossato et al., 2011).

(i) Bedrock framework. The upstream relief exposes a telescoped nappe pile. Ligurian Units, serpentinite, pillow basalts and radiolarite capped by Palombini shales (Middle–Upper Jurassic) lie tectonically over Triassic-Cretaceous carbonates of the Tuscan Unit and the Variscan basement. Calc-alkaline porphyry stocks emplaced at Gavorrano and Castel di Pietra at 5–4 Ma pierced this stack, drove vigorous hydrothermal circulation and generated the pyrite-polymetallic ore belt centred on Massa Marittima. Fluid-inclusion evidence confirms that As-rich hydrothermal quartz was eroded from these deposits and dispersed downstream (Ruggieri et al., 2021). Westwards, the structural pile is buried beneath Plio-Quaternary marine clays and estuarine-deltaic sands.
(ii) Quaternary fill and sediment dynamics. The graben-fill comprises two unconformity-bounded synthems. Synthem P1 (early–middle Pleistocene) records incision followed by aggradation of braided-stream and alluvial-fan systems that transported coarse Cr–Ni–V-rich detritus from ophiolitic source areas. Synthem P2 (late Pleistocene–Holocene) is dominated by fine-grained over-bank silts, palustrine muds and phytoclastic tufas; the combined thickness of both units reaches 80 m beneath the modern flood-plain (Costagliola et al., 2010). Present sediment delivery is governed by the flashy floods of the Pecora River and the engineered Canale Allacciante, both of which debouch across the plain before entering the Gulf of Follonica.
(iii) Metal sources and their dispersal pathways. The Massa Marittima–Campiano ore belt supplies the principal geogenic input of trace metals. Centuries of mining, followed by twentieth-century pyrite roasting, left flotation tailings and cinders on fluvial terraces, which are episodically re-mobilised during floods (Costagliola et al., 2008). Suspended loads enriched in As, Cu, Pb, Zn and Cd are conveyed along the Pecora thalweg and deposited across the industrial low land (0–20 m ASL), whereas eastern foot-slope soils (> 20 m a.s.l.) may incorporate ultramafic colluvium and receive negligible industrial fallout (Bargagli et al., 2003; Bini et al., 2017; Dini et al., 2024).

This stratigraphic-geomorphic architecture controls the hydro-sedimentary connectivity between the Colline Metallifere mining district and the coast and dictates the present spatial pattern of PTEs across the Scarlino Plain.

### Sampling and analytical determinations

The soil sampling area was identified using the Vizard et al. (2006) method within a 1.5-km radius from the industrial district centre, encompassing approximately 28 km^2^ in the Scarlino Plain, including the Casone industrial complex (Fig. 1). A total of 44 sampling sites were randomly selected using ArcGIS 9.0 (ESRI), ensuring a minimum distance of 250 m between the sites, with 11 sites in each sector (NE, NO, SO and SE). Soil sampling targeted the uppermost 0–5 cm to capture recent atmospheric deposition while minimising dilution from deeper, geogenically influenced soil layers. When sampling points fell within actively ploughed arable land or proved inaccessible or unsuitable during field surveys, samples were relocated within 250 m of the original site. In agricultural areas, relocation targeted vegetated areas along field margins positioned at least 5 m from the cultivated rows to ensure representativeness and avoid recent soil disturbance. Collaboration with the municipalities of Scarlino and Follonica, as well as with public authorities, facilitated site access and ensured efficient operations.

Superficial soil sampling was carried out in July 2014 as the first official survey requested by the provincial environmental authorities. The resulting dataset captured the pre-remediation conditions and land-use configuration that existed before any mitigation measures were implemented. The geographic coordinates of each station were logged using a handheld GPS receiver to provide precise georeferencing. This spatial accuracy makes the dataset a robust baseline for future campaigns, enabling quantitative before-and-after evaluations of remediation effectiveness and identification of new anthropogenic and geogenic PTE inputs. At each site, the spontaneous vegetation was carefully removed using disposable gloves. Turf and plant litter layers were scraped off using stainless-steel tools. Approximately 1 kg of soil was collected by carving the surface to a depth of 5 cm with a small shovel. The samples were placed in pre-labelled glass containers indicating the date, code and station name. The samples were transported to the laboratory on the same day, where large debris was removed and spread on a tray to dry in a clean room for several days. Once dried, the bulk material was sieved with a Retsch (Germany) stainless-steel set to obtain two size classes. The coarse fraction (> 2 mm) was weighed to quantify its abundance and archived; it was not subjected to further analysis. The fine fraction (< 2 mm) was homogenised, and a representative sub-sample was composited in a 250-mL polypropylene jar for subsequent chemical determinations. A separate 20 g aliquot of the same < 2 mm material was reserved exclusively for gravimetric moisture assessment: it was dried at 105 °C to a constant mass, and this aliquot was not analysed for elemental concentrations.

### PTEs analysis

Sample digestion was performed using a closed-vessel microwave digestion system (Milestone, Ethos 1, USA) equipped with 24 Teflon® (polytetrafluoroethylene) vessels and a temperature sensor for precise control. Approximately 400 mg of each powdered and homogenised soil sample (size fraction < 2 mm) was accurately weighed and digested in closed vessels under high pressure (90 bar) with 8 ml of concentrated reagent-grade HNO_3_ (Carlo Erba, Italy) and 2 ml of 30% (w/v) H_2_O_2_ (Panreac, Spain). After cooling, the mineralised solutions were transferred into polyethylene conical tubes and adjusted to 50 ml using deionised Milli-Q® water (Millipore, resistivity = 18.2 MΩ·cm^−1^). This acid digestion method (HNO_3_ + H_2_O_2_) is suitable for extracting the environmentally available fraction of trace elements (ISO, 2005). Whereas true total recoveries are normally obtained only after the very aggressive HF + HClO_4_ attack that dissolves metals locked in silicate lattices and other residual minerals, the closed-vessel microwave step with concentrated HNO_3_ + H_2_O_2_ is aimed at mobilising trace elements held in the strongly bound organic fraction and in poorly crystalline oxide or sulphide phases (Adriano, 2001). Hydrochloric acid was deliberately excluded to avoid Cl-based polyatomic interferences, which can compromise detection limits and analytical accuracy, particularly for elements such as As (Amaral et al., 2015; Link et al., 1998; Pavlíčková et al., 2003). The limited addition of H_2_O_2_ enhances the overall oxidising strength of the solution and facilitates the breakdown of organic matter (Link et al., 1998).

The concentrations of 14 PTEs (As, Cd, Co, Cr, Cu, Hg, Mn, Ni, Pb, Sb, Sn, Tl, V and Zn) were determined in each digestion aliquot using inductively coupled plasma mass spectrometry (Elan 9000, Perkin Elmer, USA). Instrument calibration was carried out for all elements using a freshly prepared aqueous multi-element reference from 1000 mg L^−1^ stock solutions (Merck, Germany). All measurements were performed in a UNI CEI EN ISO/IEC 17025-accredited laboratory. To verify analytical quality, each analytical batch included two soil certified reference materials, NIST SRM 2711a “Montana II Soil” and BCR-142R “Light Sandy Soil,” together covering certified or reference values for 13 of the 14 target elements (all except Sn). Tin, and for confirmation Tl and V, was therefore checked through matrix-matched spike additions. The mean recoveries for all elements ranged from 92 to 105%, and the relative standard deviation of triplicate digestions was always < 6%, demonstrating satisfactory accuracy and precision.

### Ecological risk assessment

The ecological risk index (RI) is a widely used metric for evaluating environmental risks associated with PTEs in soil. Developed by Håkanson (1980), the RI quantifies ecological risk by incorporating the toxicity and ecological responsiveness of individual PTEs, making it particularly valuable for assessing soil contamination in industrial and mining-affected regions. The RI for each PTE was calculated as follows:

$$RI={\sum_i}{E}_{r}^{i}={\sum_i}{T}_{r}^{i}\times {E}_{i}={\sum_i}{T}_{r}^{i}\times \frac{{C}_{i}}{{B}_{i}}$$

where.

${E}_{r}^{i}$ is the potential ecological risk factor for the *i*th element.

${T}_{r}^{i}$ represents the toxic-response factor of the *i*th PTE.

${E}_{i}$ is the contamination factor of the *i*th PTE.

${C}_{i}$ is the measured concentration of the *i*th PTE in the soil (µg g^−1^).

${B}_{i}$ is the background concentration of the *i*th PTE (µg g^−1^).

The toxic response factors (*T*_*r*_) used in this study are as follows: Sb = 7, As = 10, Cd = 30, Co = 5, Cr = 2, Mn = 1, Hg = 40, Ni = 5, Pb = 5, Cu = 5, Tl = 1, V = 2 and Zn = 1 (Doležalová Weissmannová et al., 2019; Hoshyari et al., 2023; Liu et al., 2025; Ma et al., 2020). For Sn, no *T*_*r*_ value was available; therefore, a value of 1 was assumed, based on the toxicity of similar elements. While inorganic Sn compounds are generally not highly toxic due to their low solubility and poor absorption, chemical and biochemical methylation can convert them into more toxic organic forms, such as methyltin (Ostrakhovitch, 2022).

Background concentrations (Bi) for each element were established during the preliminary impact assessments conducted in 2013 as part of the EU-mandated Environmental Impact Assessment (EIA) program described in the “Introduction.” Sampling sites were deliberately positioned in areas free from direct anthropogenic influence yet underlain by the same parent materials as the study soils to ensure geochemical comparability. We collected 10 topsoil samples (0–5 cm, < 2 mm fraction) from the metamorphic foothills east of the plain, all located > 25 m a.s.l., at least 2 km up-gradient and up-wind of the Casone industrial complex, and outside the Pecora flood-plain. These uncontaminated but lithologically equivalent soils were processed and analysed using the identical protocol applied to the main dataset, and their statistics were adopted as Bi for each PTE. The resulting background values were cross-checked against regional datasets and the relevant literature, providing a robust baseline that captures the area’s natural geochemical signature (Costagliola et al., 2008, 2010; Rossato et al., 2011; Ruggieri et al., 2021), which is crucial in a region with known geogenic anomalies, and ensuring that subsequent ecological-risk indices isolate true anthropogenic contributions. These values were as follows: Sb = 0.50 μg g^−1^, As = 8.85 μg g^−1^, Cd = 0.16 μg g^−1^, Co = 8.00 μg g^−1^, Cr = 42.6 μg g^−1^, Mn = 601 μg g^−1^, Hg = 0.10 μg g^−1^, Ni = 25.2 μg g^−1^, Pb = 13.9 μg g^−1^, Cu = 18.1 μg g^−1^, Sn = 1.65 μg g^−1^, Tl = 0.19 μg g^−1^, V = 26.2 μg g^−1^ and Zn = 50.9 μg g^−1^.

Ecological risk was classified with the Håkanson method; the four risk classes (RI < 150, 150–300, 300–600, > 600) are those originally proposed by Håkanson (Håkanson, 1980) and are still applied, without re-scaling, in recent studies that use anything from five to more than a dozen metals, e.g. 12 elements around a cement plant in Beijing (Wang et al., 2018), 11 elements in a sulphide-mineralised district (Ma et al., 2020), 7 elements around an antimony tailings pond (Zou et al., 2023) and 10 elements in an Sb-mining watershed (Liu et al., 2025). Although adding more elements will numerically increase the summed RI, the risk classes remain comparable because (*i*) the classes are anchored to toxic response factors that weight each element and (*ii*) the individual risk factors (Ei), which highlight the elements that dominate the overall index, are also reported. Therefore, this approach provides a conservative yet widely accepted assessment and keeps the results directly comparable with the international literature.

### Statistical analysis

The dataset was evaluated for normality using the Kolmogorov–Smirnov and Lilliefors tests, while homogeneity of variance was assessed using Levene’s test. Multiple normality tests were employed to provide complementary sensitivity to different aspects of distributional departures, whereas Levene’s test was selected for its robustness in assessing homoscedasticity across groups when the data may not be perfectly normal. Non-normal distributions were transformed to meet the analytical assumptions. Pairwise Pearson’s *r* coefficients between PTEs were calculated on log-transformed data, and robust statistical parameters, such as the minimum covariance determinant estimator (Reimann et al., 2008), were used to minimize the influence of outliers.

To address the compositional nature of geochemical data, concentrations were expressed in centred log-ratio (clr) coordinates, effectively removing the closure constraint that produces spurious correlations in raw compositional data (Aitchison, 1986). On these clr-transformed data (CoDaPack 2.0; Comas-Cufí and Thió-Henestrosa (2011)), principal component analysis (PCA) was applied to reduce dimensionality and identify multivariate patterns and associations in PTE concentrations across superficial soils. This approach provides orthogonal geochemical factors that are readily interpretable in terms of lithogenic versus anthropogenic inputs and yields continuous score coordinates that describe the mixed provenance of each sample, a methodology that has been successfully and increasingly adopted in several recent compositional data analysis (CoDA) studies (Ambrosino et al., 2024; Cicchella et al., 2022; Modabberi et al., 2023; Tepanosyan et al., 2020). Following the Kaiser criterion, principal components (PCs) with eigenvalues greater than one were retained and interpreted based on their loadings and relative contributions to the overall variance.

To further enhance the classification accuracy and support a clearer interpretation, K-means clustering was applied to the PCA results. The optimal number of clusters (*k* = 2) was determined by the highest average silhouette width, with supporting evidence from the gap statistic and a majority vote among several clustering validity indices, ensuring objective partitioning of the dataset. The spatial distribution of the PC loadings was visualised using kriging interpolation to reveal the contamination gradients across the study area. Exploratory data visualisation included histograms, density plots, boxplots, biplots and cluster maps, providing a comprehensive basis for interpreting multivariate relationships among PTEs and their spatial trends.

To support and validate the source attributions derived from the multivariate analysis, enrichment factors (EFs) for each PTE were computed using local background concentrations as reference values (see “Ecological risk assessment”). Spatial trends and potential source differentiation were additionally assessed through Pearson’s correlation coefficients and Theil–Sen slope estimates between the PC scores and distance from the industrial core. Finally, one-way ANOVA was used to test for significant differences in PC scores among the land-use classes. All statistical analyses were performed using R software (R Development Core Team; http://www.cran.r-project.org).

## Results and discussion

### PTE concentration in top soils

The concentrations of PTEs (As, Cd, Co, Cr, Cu, Hg, Mn, Ni, Pb, Sb, Sn, Tl, V and Zn) in soils (0–5 cm) from 44 sampling sites on the Scarlino Plain are presented in Table 1, along with the European and Italian soil baselines and screening levels. Compared to European geochemical baselines (Salminen et al., 2005), the Scarlino Plain dataset exhibited widespread enrichment of As, Mn, Cu and Zn, with average concentrations exceeding 50% above baseline values. The mean concentrations from agricultural and rural samples (*n* = 31) indicated that As and Hg levels were four and three times higher, respectively, than the baselines for Italian agricultural soils (Cicchella et al., 2015). Further comparisons with the GEMAS survey of European agricultural soils (Reimann et al., 2018), which analysed 2108 Ap-horizon samples (0–20 cm), revealed significant enrichment factors in the Scarlino soils: 3.8 for As, 1.9 for Zn, 1.6 for Cu and 1.2 for Cd. Mercury exhibited a particularly pronounced enrichment, with median concentrations nearly fourfold higher (0.11 vs. 0.03 µg g^−1^) than the European agricultural baseline. Because the baseline datasets were produced with aqua-regia extractions and deeper sampling (0–20/25 cm), whereas our study used a HNO_3_-H_2_O_2_ digestion on the 0–5-cm layer, these comparisons are intended only as broad geochemical context rather than strict regulatory equivalence.

**Table 1:** Descriptive statistics (average, standard deviation, coefficient of variation, median, minimum, maximum, 10^th^, 25^th^, 75^th^ and 90^th^ percentiles) of PTE concentrations (µg g^−1^) in the topsoil of the peri-urban area of the Scarlino Plain. Data were compared with soil contamination thresholds established by Italian legislation for residential (CSC^A^) and industrial/commercial (CSC^B^) land use and median concentrations reported by reference studies

	As	Cd	Co	Cr	Cu	Hg	Mn	Ni	Pb	Sb	Sn	Tl	V	Zn
Average	33.8	0.33	9.46	35.7	27.9	0.11	752	25.9	29.4	0.54	1.99	0.18	25.5	129
SD	35.5	0.40	4.66	14.9	18.6	0.02	786	6.21	20.8	0.05	0.63	0.07	5.99	163
CV	105	124	49.2	41.9	66.9	15.2	105	24.0	70.5	8.97	31.9	37.9	23.5	126
Median	20.7	0.22	8.4	32.3	23.3	0.11	659	26.2	21.2	0.50	1.95	0.18	26.0	83.7
Min	3.20	< 0.10	5.50	14.2	5.80	< 0.10	14.8	9.90	8.90	< 0.50	1.20	< 0.10	13.6	21.5
Max	211	2.52	36.4	101	111	0.16	5581	42.1	87.5	0.60	4.80	0.39	50.2	1066
10 perc	8.45	0.12	5.75	20.1	10.4	0.10	243	17.8	12.5	0.50	1.3	0.10	16.1	38.0
25 perc	9.43	0.15	7.23	25.9	17.8	0.1	438	22.4	14.8	0.50	1.53	0.12	21.6	52.5
75 perc	50.1	0.33	10.6	42.6	32.9	0.12	837	29.0	35.7	0.60	2.18	0.22	28.1	150
90° perc	64.6	0.53	11.9	54.1	41.2	0.14	1073	33.2	71.4	0.60	2.85	0.28	30.7	252
*n* > DL	44	35	44	44	44	29	44	44	44	11	44	41	44	44
CSC^A^ (% excess)	20 (57%)	2 (2%)	20 -	150 -	120 -	1 -	NA	120 -	100 -	10 -	1 -	1 -	90 -	150 (23%)
CSC^B^ (% excess)	50 (23%)	15 -	250 -	800 -	600 -	5 -	NA	800 -	1000 -	30 -	350 -	10 -	250 -	1500 -
European baseline^a^	7.03	0.15	7.78	60.0	13.0	0.037	382	18.0	22.6	0.60	3.00	0.66	60.4	52.0
European agricultural soil^b^	5.50	0.18	7.50	20.0	15.0	0.030	445	15.0	16.0	0.23	0.72	0.12	25.0	45.0
Italian agricultural soil^c^	7.56	0.26	11.8	33.6	32.0	0.033	664	31.5	22.1	0.32	1.03	0.18	34.2	61.7
Campania Region Survey^d^	11.7	0.3	12.1	24	86	0.07	-	22	59.9	0.52	2.9	1.4	67.7	104

^a^Topsoil, 0–25 cm ( Salminen et al., 2005)

^b^Ap horizon, 0–20 cm ( Reimann et al., 2018)

^c^Ap horizon, 0–20 cm (Cicchella et al., 2015)

^d^Campania, Southern Italy; top soil, 0–20 cm (Zuzolo et al., 2020)

**Fig. 2:**
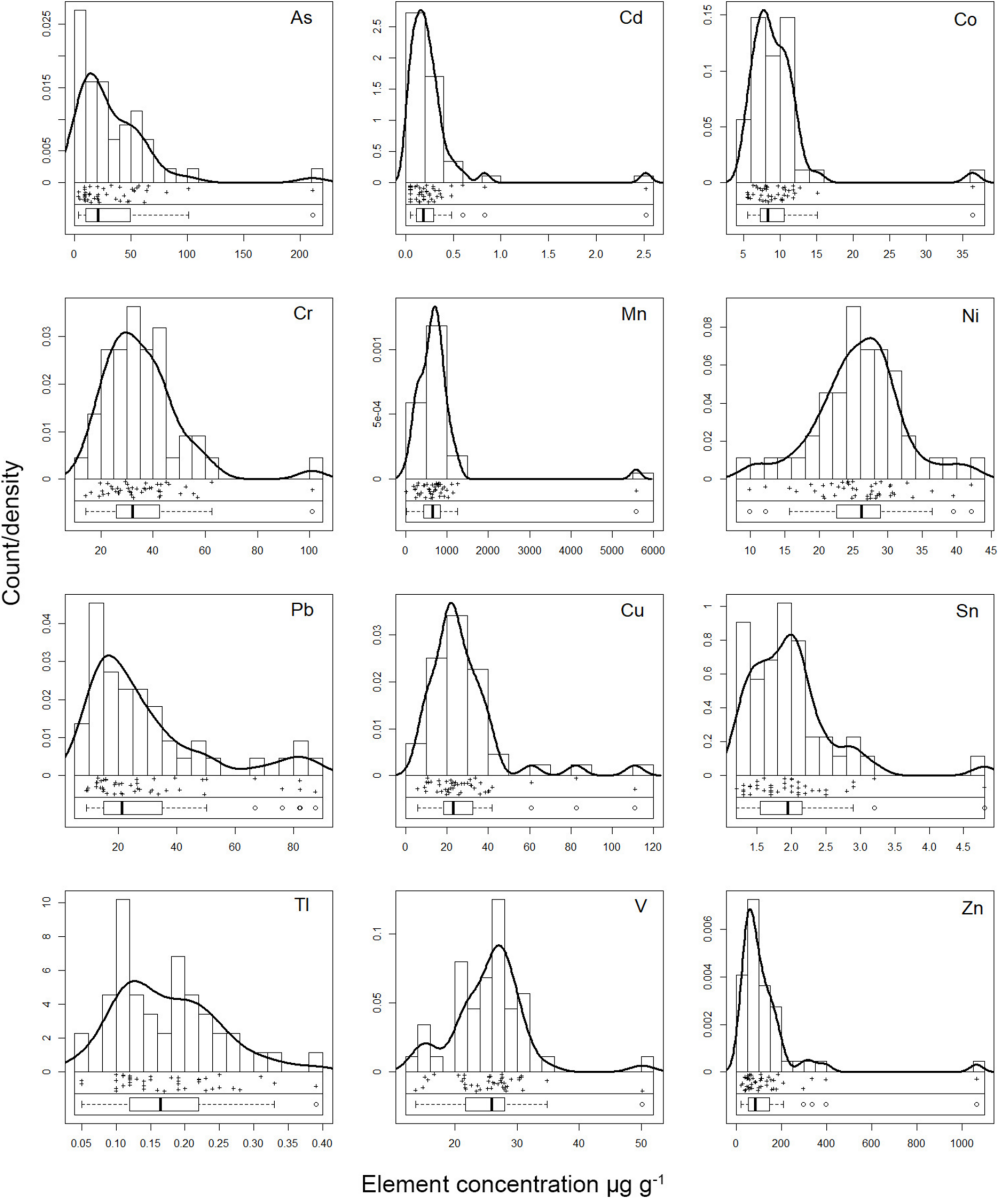
Histograms, density functions and Tukey’s boxplots of trace element concentrations (µg g^−1^) in soil (*n* = 44) from the Scarlino Plain. Hg and Sb data with DL > 30% are not shown

Except for Cr, Ni and V, all elements exhibited marked variability, with As, Cd and Zn showing distinctly skewed distributions (Table 1; Fig. 2). The distributions of As, Cd, Hg, Pb and Zn deviated from normality (Kolmogorov–Smirnov and Lilliefors tests, *p* < 0.01; Fig. 2). Arsenic (in 15 samples), Zn (in 11 samples), Cd and Co exceeded the CSC^A^ contamination threshold concentrations for public green, private and residential use (Italian Legislative Decree 152/2006). Additionally, the As concentrations at the 10 sites exceeded the CSC^B^ thresholds for commercial and industrial use. It is worth noting that these statutory limits are commonly defined for aqua-regia digests, although not explicitly specified in the legislation; therefore, exceedances detected with our slightly less aggressive method are conservative and may underestimate true concentrations relative to the legal benchmarks.

The average As concentration in superficial soils (0–5 cm) from randomly selected sites around the industrial district was 33.8 ± 35.9 µg g^−1^, nearly three times higher than the European baseline (Salminen et al., 2005). Only the 10th percentile (8.45 µg g^−1^) was aligned with normal levels. The maximum concentration (211 µg g^−1^; Site 26) occurred near a brownsite (GR66) within the “Provincial Plan for the Remediation of Polluted Areas” due to pyrite ash contamination. The highest As concentrations (> median) were concentrated between the Pecora River and the Allacciante Canal, southwest of the Casone industrial zone (Fig. 1). Urban soils showed lower As concentrations, but the average for the urban area (21.8 µg g^−1^) still exceeded the Italian regulatory screening level (20 µg g^−1^) for residential zones (CSC^A^). Lower concentrations (<  1 st quartile = 16.4 µg g^−1^) were found mainly at the eastern sites of the canal, particularly in the southeastern foothills.

**Fig. 3:**
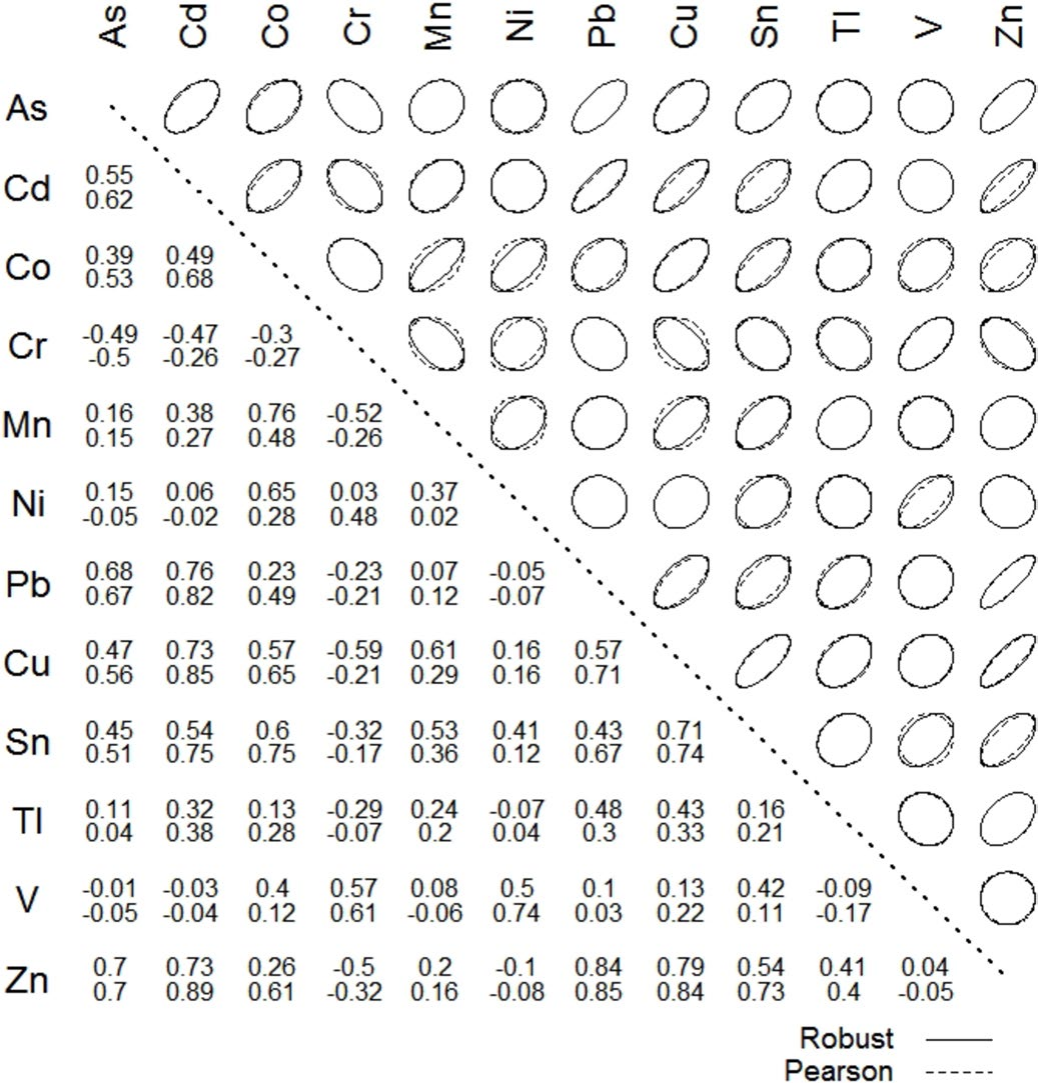
Correlation coefficients and correlation ellipses on log-normalised data of element concentrations in superficial soils of the Scarlino Plain. Upper number: robust minimum covariance determinant (MCD; Reimann et al., 2008); lower number: Pearson’s correlation coefficient. Hg and Sb were excluded from the correlation matrix due to detection limits > 30%

**Fig. 4:**
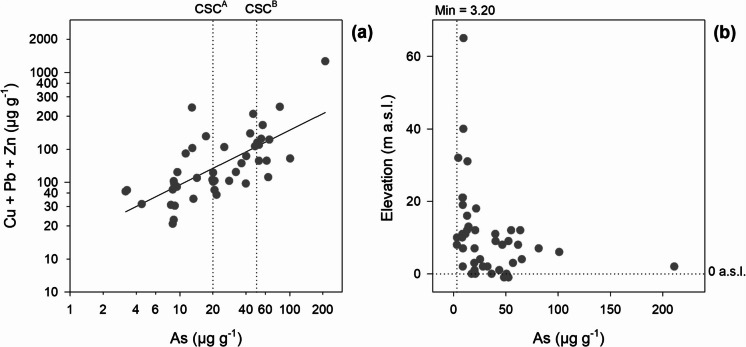
**a** As concentration (µg g⁻^1^) plotted against the sum of Cu, Pb and Zn concentrations (µg g⁻^1^; indicator of pyrite-related contamination) on a logarithmic scale. Horizontal dashed lines indicate the Italian soil contamination thresholds for residential (CSC^A^) and industrial/commercial (CSC^B^) land use. **b** As concentration (µg g⁻^1^) plotted against elevation of sampling sites (m a.s.l.), showing higher concentrations predominantly below 18 m

Arsenic showed a positive correlation with Cd, Cu, Pb and Zn (*p* < 0.01; Fig. 3), indicative of the presence of sulphide mineralisation, commonly associated with pyrite in ore deposits. The weathering of pyrite and related sulphide minerals, particularly after roasting and exposure to environmental conditions, releases these metals into the surrounding soil, increasing their concentrations. When the As concentrations were plotted (Fig. 4a) against the sum of Cu, Pb and Zn concentrations on a logarithmic scale, a positive correlation was observed, suggesting that the As contamination originated from pyrite-related processes. This trend was most pronounced in soils exceeding the Italian screening level for As contamination (20 µg g^−1^), predominantly found at lower elevations (< 18 m a.s.l.) in the plain valley basin, particularly in industrial zones heavily impacted by pyrite-derived contamination (Fig. 4a). In contrast, soils with lower As concentrations (including the minimum of 3.20 µg g^−1^) were commonly found at higher elevations (i.e. > 20 m a.s.l.; Fig. 4b). The spatial distribution of As reflects both natural processes, including sediment transport and geogenic inputs, and anthropogenic sources, such as atmospheric deposition from pyrite roasting and historical industrial waste disposal, which collectively determine the contamination patterns in the Scarlino Plain (Rossato et al., 2011).

Cadmium in soil can disrupt microbial communities, reducing their diversity and function, which is essential for soil health. Its bioavailability is also a concern, as Cd can be readily absorbed by plants and potentially accumulate in the food chain (Li et al., 2023). In the study area, Cd concentrations averaged 1.5 higher than the European baseline and were consistent with levels in industrially influenced areas in Northern Tuscany (Bretzel & Calderisi, 2006). Ninety percent of the sites had Cd concentrations below 0.53 µg g^−1^, with elevated values (0.60 and 0.83 µg g^−1^) detected at only two stations near the Canale Allacciante. The maximum concentration recorded at Site 26 (2.52 µg g^−1^) exceeded the average (0.33 µg g^−1^) by an order of magnitude. Additionally, Cd concentrations in 20% of the samples were below the limit of quantification (LOQ, 0.1 µg g^−1^).

Cobalt, an essential trace element, poses significant ecological and health risks when present in excess in the soil. Its accumulation in agricultural areas can lead to its entry into the food chain, causing toxic effects in plants, animals and humans (Srivastava et al., 2022). In the study area, Co concentrations were generally consistent with continental-scale reference values and aligned with other datasets from the Italian territory (Cicchella et al., 2015; Zuzolo et al., 2020; Table 1). Most values fell below 11.9 µg g^−1^ (90th percentile); however, elevated concentrations were recorded at three industrial stations, notably site 26, which exhibited a peak value of 36.4 µg g^−1^. In addition to traditional anthropogenic sources, such as metallurgical activities, Co contamination may also result from titanium dioxide (TiO_2_) production processes, which generate waste containing trace levels of Co (Xu et al., 2024).

Chromium contamination in soil and groundwater has raised significant concerns due to anthropogenic contributions, particularly from industrial activities, even in regions where natural sources of Cr exist. Urban areas often show elevated Cr levels near roads and agricultural fields, reflecting historical land use (Bretzel & Calderisi, 2006). In Tuscany, Cr concentrations varied significantly and were influenced by both geogenic and anthropogenic factors. High Cr levels in the Cecina Valley are mainly geogenic, linked to ophiolitic materials and ultramafic rock weathering (Tassi et al., 2018). In contrast, industrial areas, such as those near tanneries, exhibit extreme Cr concentrations of up to 10,000 µg g^−1^ (Bini et al., 2008). In our study, the average Cr concentration matched the European average reference, with most values (1st–3rd quartiles) ranging narrowly between 25.9 and 42.6 µg g^−1^. The highest concentration (101 µg g^−1^) was observed near the industrial/commercial area of Follonica. These findings are consistent with the typical background for the region and the continent: in coastal-Tuscany urban soils. Bretzel and Calderisi (2006) measured a mean total Cr concentration close to 35 mg kg⁻^1^, while the pan-European survey of top soils reported a median of 60 mg kg⁻^1^ for 845 sites (range < 3–6230 mg kg⁻^1^) (Salminen et al., 2005). Additionally, strong positive correlations were observed between Cr, Ni and V (*p* < 0.01; Fig. 3), suggesting a shared source or similar geochemical behaviour, likely linked to ophiolitic or ultramafic rocks, which are known to be natural reservoirs of these elements in the region (Bargagli et al., 2003; Bini et al., 2017; Dini et al., 2024).

Copper contamination is commonly attributed to pesticide use in vineyards and orchards and is influenced by climate, agricultural history and soil organic matter (Neaman et al., 2024). In coastal Tuscany, Cu concentrations reached 300 µg g⁻^1^ in agricultural soils due to prolonged pesticide application in vineyards (Bretzel & Calderisi, 2006), consistent with findings across Italy (De Bernardi et al., 2022). In the study area, Cu concentrations exhibited moderate variability (CV = 66.9%), with a mean of 27.9 µg g⁻^1^ and a median of 23.3 µg g⁻^1^. Most values were below the 90th percentile (41.2 µg g⁻^1^), indicating a generally moderate distribution, although several elevated concentrations were observed at urban and industrial sites (60.9, 82.5 and 111 µg g⁻^1^). The minimum value (5.80 µg g⁻^1^) was recorded east of the Allacciante Canal (Fig. 1). Despite the presence of high values, the average Cu concentration was more than twice the European baseline (13 µg g⁻^1^; Salminen al. 2005), yet consistent with the Italian agricultural baseline (32 µg g⁻^1^; Cicchella et al., 2022). The spatial pattern suggests contributions from airborne industrial emissions and historical pyrite smelting (Sun et al., 2023).

Industrial activities and pyrite weathering are significant sources of Hg contamination in soils, which are influenced by environmental conditions such as pH, the presence of oxidants and microbial activity. The interaction between Hg and sulphur species is critical in these processes, as it affects Hg mobility and bioavailability (Manceau et al., 2018). In the Scarlino Plain, 34% of the Hg concentrations were below the LOQ, while the remaining values ranged narrowly between 0.10 and 0.16 µg g^−1^. The average Hg concentrations exceeded the baseline levels reported in the literature (Table 1) but remained within the European guidelines, including the Dutch target value of 0.3 µg g^−1^. Notably, most soil samples (*n* = 3) showed Hg levels that were approximately one order of magnitude higher than the baseline. The Hg concentrations in Scarlino were significantly lower than those in other areas of Tuscany. For example, past operations at the Rezzaio plant, where barite and iron oxide ores (hematite and magnetite) were roasted and processed, led to soil Hg concentrations as high as 88 μg g^−1^ (Nisi et al., 2024). In urban Pisa, leather manufacturing led to concentrations as high as 170 μg g^−1^, raising potential non-carcinogenic health risks, particularly in children (Ghezzi et al., 2022). The former Hg mining area of Abbadia San Salvatore in southern Tuscany exhibited extreme contamination, with soil concentrations reaching 1068 μg g^−1^ (Meloni et al., 2023).

Nickel contamination in soils poses significant risks to soil health, plant growth and microbial communities, impacting ecological processes and potentially threatening food safety and human health (Zhang et al., 2022). At high concentrations, Ni is toxic to plants, impairing growth and physiological functions (Rizwan et al., 2024). In Scarlino, the Ni concentrations followed a normal distribution, with mean and median values nearly identical to and below the European soil reference (Table 1). The data exhibited one of the lowest coefficients of variation, indicating minimal variability.

Manganese concentrations in the study area ranged from 14.8 to 5581 μg g^−1^ (Site 26), with 90% of the data falling below 1000 µg g^−1^. The average Mn concentration was 33% higher than the European reference level (Table 1). Mn contamination is often associated with industrial processes and can lead to widespread soil pollution in industrialised regions. This contamination has various ecological impacts, including potential toxicity to soil organisms and alterations in soil chemistry (Herndon et al., 2011). In the Scarlino Plain, historical industrial activity involving the production of TiO_2_ from ilmenite using sulphuric acid likely contributed to localised Mn enrichment. This process involves the leaching of Mn compounds during acid digestion of the mineral, which can contaminate the environment if not properly managed (Jabłoński & Tylutka, 2016).

The average Pb concentration in the soil of the Scarlino Plain was lower than the European reference values. Most sites (75%) had Pb concentrations below 36 µg g^−1^, with particularly low values (9.5 and 24.2 µg g^−1^) observed east of the Allacciante Canal. Extreme values above 80 µg g^−1^ were recorded at the three urban and industrial sites, while moderately high concentrations (48.8–76.1 µg g^−1^) were identified in the southern area (Fig. 1). The presence of Pb in these soils is mainly attributed to the geological substrate and historical mining activities, particularly pyrite extraction and processing, which released Pb-bearing minerals such as galena (PbS) into the environment (ARPAT, 2015). Given that galena frequently co-occurs with pyrite in ore bodies, it is plausible that it was unintentionally co-processed during mineral separation, especially under the inefficient metallurgical techniques typical of earlier operations. Consequently, residual Pb-bearing waste may have contributed to localised contamination, consistent with findings from other industrial and mining-impacted regions (Doležalová Weissmannová et al., 2019; Zou et al., 2023).

In this study, 75% of the soil samples had Sb concentrations below the detection limit of 0.5 µg g⁻^1^. At detectable levels, Sb concentrations showed minimal variation across the study area (CV < 10%) and remained consistently below the values reported in the literature (Table 1). Baseline Sb concentrations in surface soils are typically low (0.06–0.79 µg g⁻^1^) and can vary significantly due to local geological and anthropogenic factors. For example, in the Berlin Metropolitan Area, Sb concentrations range from 0.54 µg g⁻^1^ in forest soils to 1.75 µg g⁻^1^ in roadside soils, reflecting a 2–6 times enrichment over the regional background value of 0.3 µg g⁻^1^ (Thestorf & Makki, 2021).

The average Sn concentration in the Scarlino area was less than half of the European values reported by Salminen et al. (2005). Tin levels exhibited low variability (CV = 34%) and predominantly ranged from 1.5 to 2.2 µg g^−1^, with the highest concentration recorded at industrial site 26. While Sn contamination in soils can have significant environmental impacts, these effects are mainly associated with regions of intensive Sn mining, such as the Bangka Belitung Islands in Indonesia and parts of Southwest China (Liu et al., 2021).

Thallium is a highly toxic heavy metal that accumulates in agricultural products and poses significant health risks to humans (Sun et al., 2023). In soils, it co-migrates with elements such as As, Fe, S, K and Rb, reflecting complex interactions within soil matrices (Yuan et al., 2024). In the Scarlino Plain, Tl concentrations consistently remained below 0.4 µg g^−1^, lower than the European reference levels (0.66 µg g^−1^). The highest concentration (0.39 µg g^−1^) was recorded at site 26.

The primary natural source of V in soils is the parent material, with concentrations varying according to geological origin. Natural V levels in soils typically range between 23 and 58 µg g⁻^1^, with ophiolitic rocks identified as a significant source (Petrini et al., 2022). However, soils can also be enriched with V through anthropogenic activities, including industrial emissions, mining and wear of vehicle brake linings and tires (Li et al., 2023). In this study, the V concentrations exhibited low variability (CV = 22.7%), suggesting a normal distribution with homogeneous values significantly lower than the European reference levels (Table 1). The highest V concentration was recorded in the Padule wetland area (Fig. 1).

Zinc is a highly abundant metal in soil, with natural concentrations ranging from 10 to 300 µg g^−1^ and a mean of approximately 50 µg g^−1^ (Kaur et al., 2024). In the Scarlino Plain, the average Zn concentration (108 µg g⁻^1^) was notably higher than the reference levels (Table 1), even after excluding the extreme value from site 26, where the concentrations exceeded the median by over 12 times. Additional high Zn concentrations (> 75th percentile, 150 µg g⁻^1^) were recorded, ranging from 153 to 396 µg g⁻^1^. These elevated levels were distributed across a broad area of the study region spanning the northeast and southwest sectors.

### Multivariate spatial analysis and sources of PTE contamination

Prior to interpretation, we verified that the dataset met the assumptions for factor analysis. Sampling adequacy was “meritorious”, with a Kaiser–Meyer–Olkin index of 0.78, and Bartlett’s test of sphericity strongly rejected the null hypothesis of an identity correlation matrix (*χ*^2^ = 412, df = 91, *p* < 0.001; Table S1, Supplementary material). The principal component analysis (PCA) applied to the soil PTE concentration dataset identified four principal components (PCs) that collectively explained 77.0% of the original total variance. The scree plot showed a clear inflexion at the fourth eigenvalue (Fig. S1, Supplementary material), supporting the retention of four principal components (eigenvalues > 1) that together explained 77% of the variance. This high percentage demonstrates the effectiveness of PCA in reducing dataset dimensionality while preserving critical information. Moreover, the leave-one-out cross-validation of the clr-PCA model yielded an RMSE of 4.6%. A Hotelling’s *T*^2^ vs. *Q* residual plot (Fig. S2, Supplementary material) identified five samples beyond the 95% confidence boundary which can be considered outliers. These diagnostics still support that the retained PCs offer a stable and parsimonious representation of the PTE dataset.

**Fig. 5:**
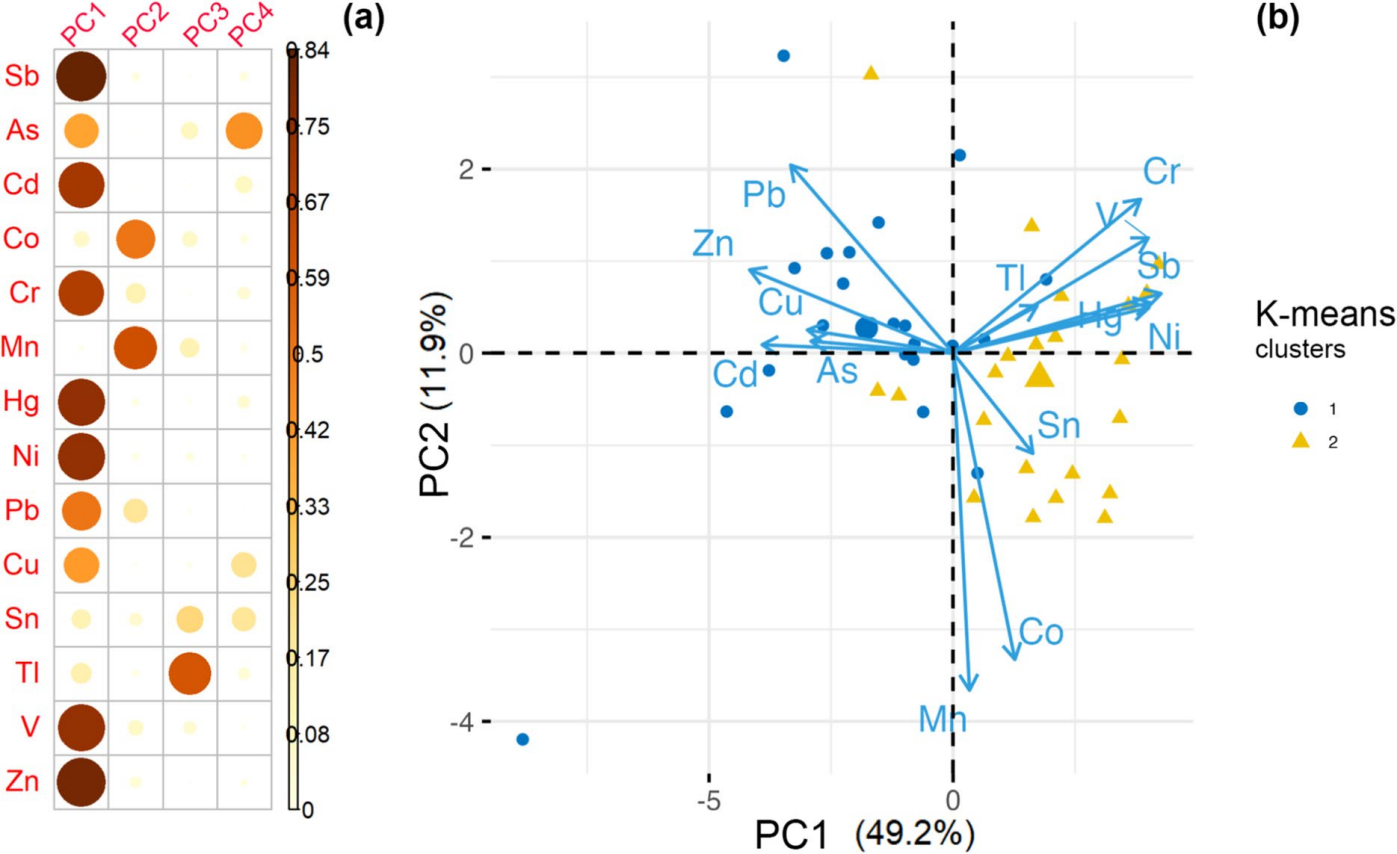
**a** Matrix of squared factor loadings of variables on the first four principal component axes. **b** PCA biplot of PC1 (49.2 % variance) vs. PC2 (11.9 %) showing the 43 soil samples classified by k-means clustering (k = 2) based on their clr-PCA scores. The points are coloured and shaped according to the cluster membership

Principal component analysis reduced the multivariate dataset into four orthogonal factors that collectively characterised the behaviour of PTEs (Fig. 5a; Table S2, Supplementary material).

PC1 (49.2% of the total variance) exhibited strong positive loadings for As, Cd, Cu, Pb and Zn (≥ 0.70) and strong negative loadings for Cr and Ni (≤ − 0.80). This chalcophile-rich and lithophile-poor contrast reflects the dispersion of pyrite-roasting residues and tailings. Similar element associations have been documented in former pyrite-mining districts in Jingmen (Ma et al. 2019) and Laizhou (Cao et al., 2024) as well as in Swedish acid-sulphate soils where Fe-oxyhydroxides control the mobility of Cd, Cu, Pb and Zn in the presence of residual pyrite (Tiberg et al., 2017).

PC2 (12.5% variance) was characterised by high loadings of Mn (0.76) and Co (0.73), with moderate contributions from V (0.52) and Ni (0.44). These elements are typical of mafic–ultramafic source rocks exposed upstream, with their concentrations further enhanced by reductive dissolution of Mn oxides during seasonal water-logging of terrace soils. PC2 thus represents a combined geogenic–pedogenic signal resulting from the erosion of ophiolitic lithologies and subsequent redox-mediated redistribution in the floodplain.

PC3 (eigenvalue = 1.14; 8.8% variance) displayed pronounced negative loadings for Tl (− 0.77) and Sn (− 0.53). High PC3 scores were concentrated around the industrial precinct and major road network, suggesting diffuse technogenic sources, including tire and brake wear, combustion of Sn-bearing alloys and regional fallout of industrial particulates (Liu et al., 2021; Sun et al., 2023).

PC4, as well as components with eigenvalues < 1, accounted for only minor portions of variance and were not interpreted further. Squared factor loadings, which represent the quality of representation of each element on the retained axes, are presented in Fig. 5a.

The PCA biplot (Fig. 5b) illustrates the relationships between PTEs and soil samples clustered into three k-means groups. The k-means partition of PCA scores (*k* = 2; Fig S3, Supplementary material) clearly separated an industrial plume cluster enriched in chalcophile elements (Pb, Zn, Cu, Cd and As; cluster 1) from a lithogenic cluster dominated by mafic-affine elements (Cr, Ni, V, Mn, Co; cluster 2). Their geographical distribution mirrors the chemical trends: cluster 1 is concentrated within ~ 1.5 km of the former Casone pyrite-roasting complex, whereas cluster 2 occurs farther away in areas with distinct lithological characteristics. This pattern corroborates the dual origin of contamination, with industrial fallout overlying the natural lithogenic background (Table S3, Supplementary material).

**Fig. 6:**
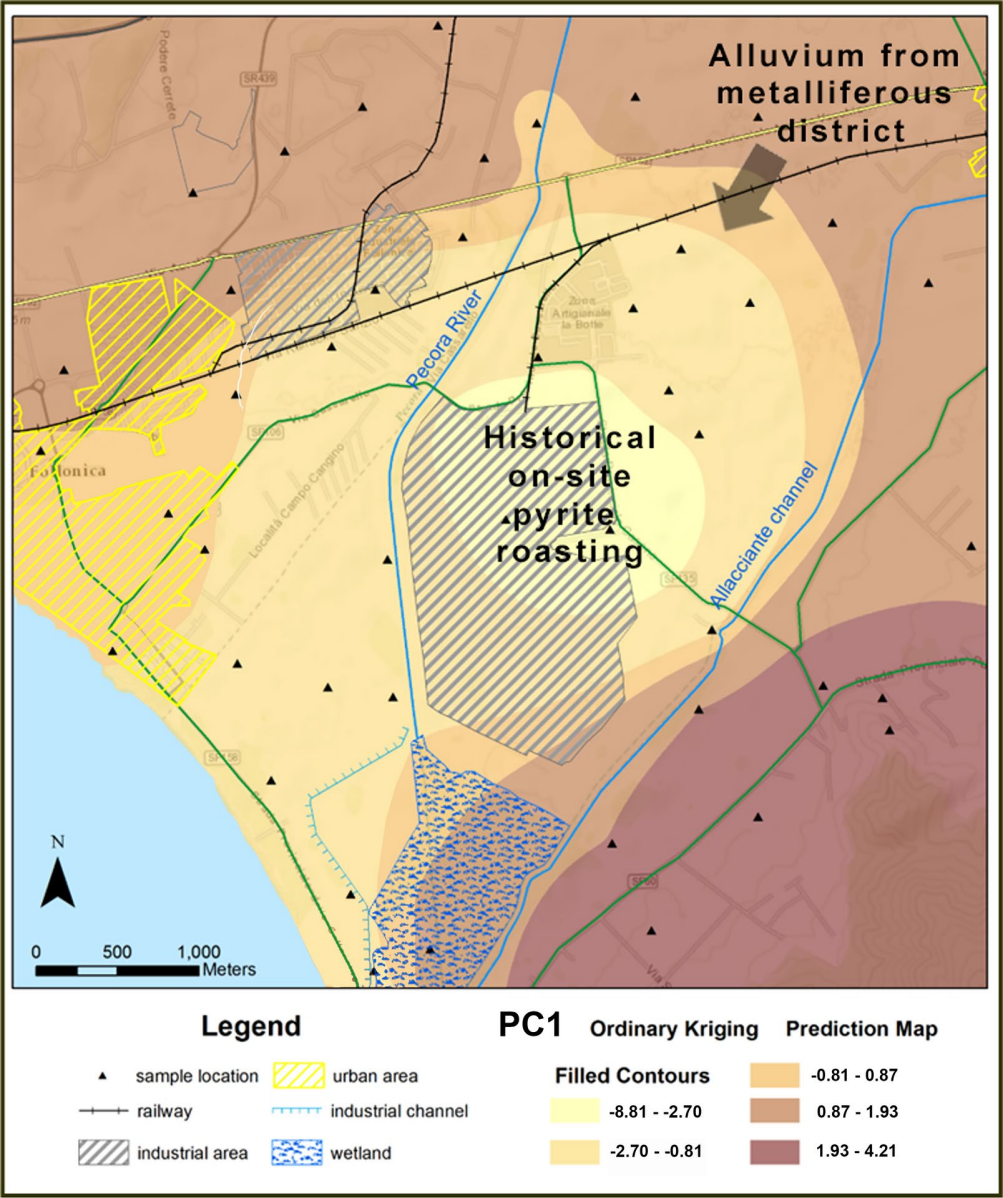
Spatial distribution map of PC1 scores representing the contribution of pyrite load to soil elemental composition at the 44 sampling sites. The map shows the kriging-interpolated contour levels, highlighting areas with varying pyrite-related elemental concentrations

PC1 emerged as a robust synthetic indicator of contamination patterns in the Scarlino Plain, delineating the interplay between natural geogenic sources and anthropogenic inputs. The spatial distribution of PC1 loadings, as presented in Fig. 6, highlights the areas affected by pyrite-related PTEs. This contamination landscape is shaped by a combination of natural geochemical anomalies and historical industrial activities.

The geological framework of the Colline Metallifere region contributes to naturally high PTE. Weathering of ore deposits in the upstream highlands has resulted in PTE-rich sediments being transported to and deposited in low-lying areas, such as the Scarlino Plain, which serves as a sediment trap (Costagliola et al., 2004, 2008, 2010). Industrial activities, particularly historical pyrite roasting for sulphuric acid production, have further elevated local concentrations of As, Cd, Pb and other PTEs. Figure 6 depicts the historical pyrite roasting sites and metalliferous sediment sources, illustrating the origins of contamination in the area. Legacy contamination extends beyond the industrial zones, forming concentration gradients that affect primarily agricultural areas, particularly in the northeastern and eastern sectors, and to a lesser extent, adjacent urban zones. In recent decades, activities that have been subsequently added, such as waste incineration and chemical manufacturing, have contributed additional PTE inputs, exacerbating historical contamination (ARPAT, 2015). The cumulative impact of geogenic and anthropogenic sources presents a unique challenge for environmental management. Distinguishing between the natural background levels and anthropogenic elevations is essential for setting realistic remediation targets (Reimann et al., 2009).

To validate the source assignments derived from multivariate analysis, we conducted three complementary analyses. First, enrichment factors (EFs) calculated against local background values (Table S2, Supplementary material) revealed strong enrichment of As (mean EF ≈ 10.7) and Zn (mean EF ≈ 9.4) at sites with high PC1 scores, whereas Cr (mean EF ≈ 0.58) and Ni (mean EF ≈ 0.94) remained at or below background levels, confirming PC1’s association with non-geogenic inputs. PC1 scores increased log-linearly with increasing distance from the former roasting complex (Pearson’s *r* =  + 0.64, *p* < 0.001; Theil–Sen slope =  + 9.55 log-units per km, *p* < 0.001; Fig. S4, Supplementary material), reflecting the exponential decay in smelter-derived metal burdens observed at comparable pyrite-roasting and smelting sites (Anaman, 2024; Wei et al., 2009; Zeng et al., 2022). Third, one-way ANOVA showed that industrial plots had markedly higher PC1 scores than agricultural or urban soils (*F* = 12.3, *p* < 0.001), whereas the Mn–Co-dominated PC2 did not differ among land-use classes (*p* = 0.55), indicating that this factor likely reflects a predominantly geogenic signal associated with the regional geochemical background (Salminen et al., 2005).

### Ecological risk assessment of soil PTEs

**Fig. 7:**
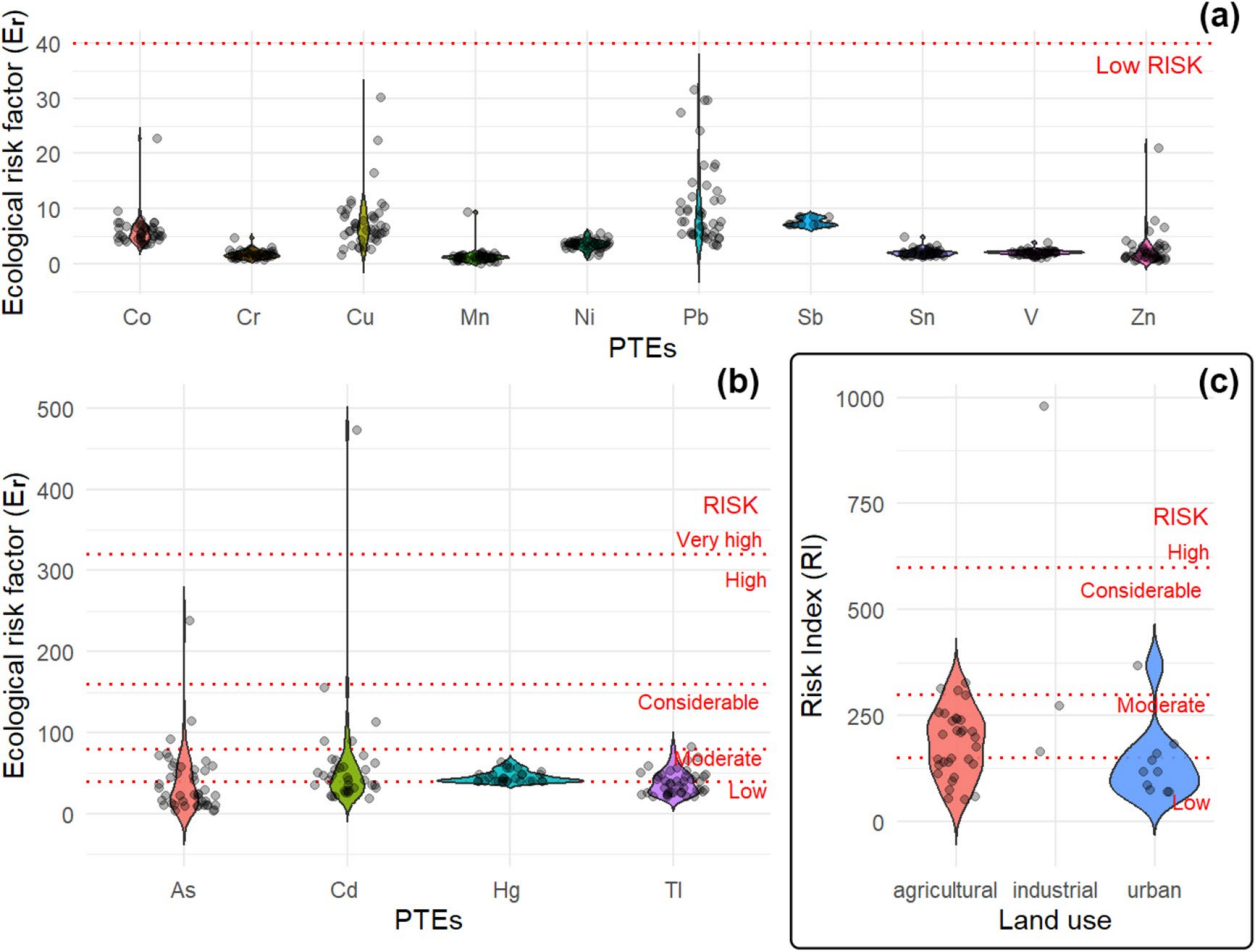
Ecological risk factors (*E*_*r*_) for PTEs in soils across the study area grouped as low-risk (Co, Cr, Cu, Mn, Ni, Pb, Sb, Sn, V and Zn) **a** or higher-risk elements (As, Cd, Hg and Tl) **b**. Risk Index (RI) distribution across different land-use types (agricultural, industrial and urban) **c**. Risk thresholds are indicated by dashed red lines in Håkanson’s (1980) contextual interpretation of risk severity

Figure 7 illustrates the distribution of ecological risk factors (E_r_) and the cumulative Risk Index (RI) across PTEs and land-use types in our study area, identifying the critical areas of concern. Most PTEs fall within the “low” or “moderate” risk categories; however, certain elements, particularly As and Cd, reach “considerable” and “high” risk thresholds, especially in industrial and agricultural zones. For Cd specifically, the elevated risk assessment was notably influenced by an extreme outlier which exceeded the dataset mean by an order of magnitude. Notably, peri-urban agricultural soils consistently demonstrated moderate to considerable cumulative risk indices (Fig. 7c), underscoring the combined influence of natural geogenic sources and historical human activities inherent to the geochemical context of the area. Although the ecological risk index was originally developed for aquatic systems, where element bioavailability is typically higher (Håkanson, 1980), its application to soils has been validated through numerous studies (Doležalová Weissmannová et al., 2019; Liu et al., 2025; Ma et al., 2020; Wang et al., 2018; Zou et al., 2023). Nevertheless, we acknowledge that soil-specific factors, including reduced element mobility, complex sorption mechanisms and variable pH conditions, may influence the absolute risk values, and our results should be interpreted considering these methodological limitations.

Our findings partially align with those of prior studies on peri-urban environmental risks, which often highlight industrial and traffic-related emissions as the dominant contributors (Hoshyari et al., 2023; Wang et al., 2022; Zou et al., 2023). However, our results highlight ecological risks in agricultural soils, consistent with previous studies reporting significant accumulation of PTEs, particularly Tl, Hg and As, in farmlands located near mining areas. These accumulations have been attributed to legacy mining activities and geochemical dispersion (González et al., 2011; Ma et al., 2020). Li et al. (2014) similarly identified As and Cd as priority contaminants, aligning with our findings from the Scarlino Plain.

The ecological risks posed by As and pyrite-related trace element contamination in agricultural soils near mining and metallurgical areas require thorough attention. These soils act as critical interfaces between natural and human-influenced systems and require integrated management strategies to mitigate the associated risks. As highlighted in previous studies (González et al., 2011; Robson et al., 2013), effective management should encompass (*i*) comprehensive soil quality assessments, including the speciation and mobility of PTEs, to understand the actual risks and inform remediation strategies; (*ii*) regular monitoring of contamination levels and bioavailability of PTEs, tracking changes over time and assessing the effectiveness of mitigation measures; (*iii*) implementation of soil remediation techniques, wherever possible, to reduce bioavailability, such as immobilising contaminants through amendments that stabilise toxic metals and (*iv*) land-use planning, limiting or restricting agricultural activities in highly contaminated areas to prevent exposure to toxic elements.

Moreover, Fig. 7 emphasises the urgent need for site-specific interventions in areas classified as “considerable” or “high” risk. International reference standards, including Canadian and Dutch guidelines (CCME, 2001; VROM, 2000), establish 11 μg g⁻^1^ in the < 2 mm soil fraction as a conservative threshold for preliminary contamination assessment prior to health risk evaluation. Our results revealed substantial exceedances of this benchmark in the Scarlino Plain surface soils. While As concentrations in the study area ranged from 32 to 82 μg g⁻^1^, which are within the ecological risk thresholds reported by Huang et al. (2024) for different types of soils, the risks associated with As can manifest even at lower concentrations. Schmidt (2014) noted the absence of a clear safety threshold for As, as even minimal exposure can disrupt soil health, plant growth and microbial communities. These findings emphasise the importance of stringent regulatory standards and proactive measures to protect both the environment and human health in regions affected by such contamination.

## Conclusions

This study demonstrates the complex interplay between geogenic and anthropogenic sources in determining PTE contamination patterns in historically industrialised regions. In the Scarlino Plain, elevated As concentrations exceeding regulatory thresholds (20 μg g^−1^) were detected in 57% of the surface soil samples (0–5 cm). Principal component analysis effectively separated pyrite-related anthropogenic contamination from natural geochemical signatures, with PC1 capturing the spatial distribution of industrial legacy pollution. Ecological risk assessment, though based on the Håkanson method originally developed for aquatic systems and later broadly adapted to soils, identified As and Cd as the main contributors to elevated risk indices, particularly in agricultural areas, where cumulative risks were highest. Because the concentrations reported here were obtained with a HNO_3_–H_2_O_2_ digestion rather than the aqua-regia procedure adopted in continental baseline datasets (e.g., FOREGS; GEMAS), direct numerical comparisons, particularly for lithophile elements, should be interpreted with caution in view of the element-specific extraction efficiencies of the two methods.

Our findings revealed a distinctive contamination profile characterised by strong correlations among As, Cd, Cu, Pb and Zn, reflecting the co-occurrence of natural geochemical anomalies and anthropogenic inputs from historical pyrite processing activities. This contamination exhibits clear spatial patterns influenced by local topography and historical land use, with the most severe impacts concentrated in low-lying areas between the Pecora River and the Allacciante Canal. Compositional PCA successfully segregated anthropogenic components (As, Cu, Cd, Pb, Zn) dominating within 1.5 km of the industrial district from geogenic components enriched with Cr, Ni and V.

The application of locally derived background values, rather than generic thresholds, enabled the accurate identification of discrete hotspots where targeted mitigation would yield maximum benefits. This approach is essential for disentangling industrial from natural contributions, thereby establishing realistic remediation goals that safeguard agricultural productivity and human health. The risk-based framework developed in this study offers a transferable methodology for legacy contamination assessment in peri-urban soils, providing decision-makers with evidence-based tools to prioritise remediation under the EU Soil Strategy for 2030, while advancing sustainable land management in historically industrialised regions.

## Supplementary Information

 Below is the link to the electronic supplementary material.

Supplementary file1 (PDF 495 KB)

## Data Availability

The dataset generated, used, and analysed in this study is available from the corresponding author upon reasonable requests.
