# Supplementary material for: Spatial distribution and ecological risk of potentially toxic elements in peri-urban soils of a historically industrialised area
Source: Environ Monit Assess. 2025 Jul 24;197(8):948. doi: 10.1007/s10661-025-14389-5 (PMC12289831; doi:10.1007/s10661-025-14389-5)
Supplement: Supplementary file 1 — Supplementary file1 (PDF 495 KB) [file 10661_2025_14389_MOESM1_ESM.pdf]

Supplementary material

Spatial distribution and ecological risk of potentially toxic elements in peri-urban soils of a historically industrialised area

Fabrizio Monaci[1,2]* and Davide Baroni[3]

[1] Department of Life Science, University of Siena, Via A. Mattioli 4, Siena 53100, Italy; fabrizio.monaci@unisi.it

[2] National Biodiversity Future Center (NBFC), 90133 Palermo, Italy

[3] Department of Environmental, Earth and Physical Sciences, University of Siena, Via A. Mattioli 4, Siena 53100, Italy; davide.baroni@unisi.it

* Correspondence: fabrizio.monaci@unisi.it; Tel.: +39 0577 232074.

Content

Table S1. Pre-PCA diagnostic statistics for the Scarlino Plain soil PTE dataset: overall Kaiser–Meyer–Olkin (KMO) measure of sampling adequacy (MSA), Bartlett's test of sphericity (χ^2, degrees of freedom, p-value), and individual MSA values for each potentially toxic element. 2

Fig. S1. Variance explained by principal components in the PTE dataset. PC1 accounts for nearly 50% of the total variance, highlighting its dominant role in capturing contamination patterns. 2

Fig. S2. Scatterplot of Hotelling's T^2 against Q-residuals for all 44 soil samples, using the first two principal components of the CLR-transformed PTEs concentrations. The dashed ellipse marks the 95 % confidence boundary (Mahalanobis distance), and the five samples falling outside that region are highlighted in red as outliers. 3

Table S2. Principal component (PC) loadings for the retained components (eigenvalue > 1) at each sampling site in the Scarlino Plain, showing the correlation (factor) loadings of each PTEs on each PC together with the percent variance explained by each component; and the corresponding enrichment factors (EFs) for each element, calculated relative to the local soil baseline concentrations. 4

Fig S3. Average silhouette width as a function of the number of k-means clusters (k) applied to the centred log-ratio–transformed PTE concentration data. The dashed vertical line at k = 2 highlights the optimal clustering solution, corresponding to the highest mean silhouette width. 5

Table S3. Summary of the two k-means clusters derived from centered log-ratio PCA of potentially toxic element (PTE) concentrations in the Scarlino Plain soils. For each cluster, the table reports: (1) the symbol/colour used in Fig. 5b; (2) the mean PC1 and PC2 scores; (3) the dominant metals identified by significant z-clr loadings; (4) the average distance of sites in the cluster from the former Casone pyrite-roasting plant; and (5) the inferred source signature for each cluster. 5

Fig. S4. Relationship between PC1 scores and $\log_{10}$-transformed distance from the former Casone pyrite-roasting complex. PC1, dominated by As, Cd, Cu, Pb, and Zn, decreases with smelter influence. Theil–Sen regression (dashed line) and Pearson's $r = 0.64$ ($p < 0.01$) indicate an exponential decline in PTEs enrichment with distance. 6

Table S1. Pre-PCA diagnostic statistics for the Scarlino Plain soil PTE dataset: overall Kaiser–Meyer–Olkin (KMO) measure of sampling adequacy (MSA), Bartlett's test of sphericity (χ^2, degrees of freedom, p-value), and individual MSA values for each potentially toxic element.

Statistic	Value
Overall KMO (MSA)	0.78
Bartlett's test of sphericity χ^2	412.00
Degrees of freedom (df)	91
p-value	< 0.001
**Variable**	**MSA (KMO per variable)**
Sb	0.64
As	0.72
Cd	0.81
Co	0.68
Cr	0.75
Mn	0.70
Hg	0.66
Ni	0.74
Pb	0.73
Cu	0.79
Sn	0.65
Tl	0.62
V	0.71
Zn	0.77

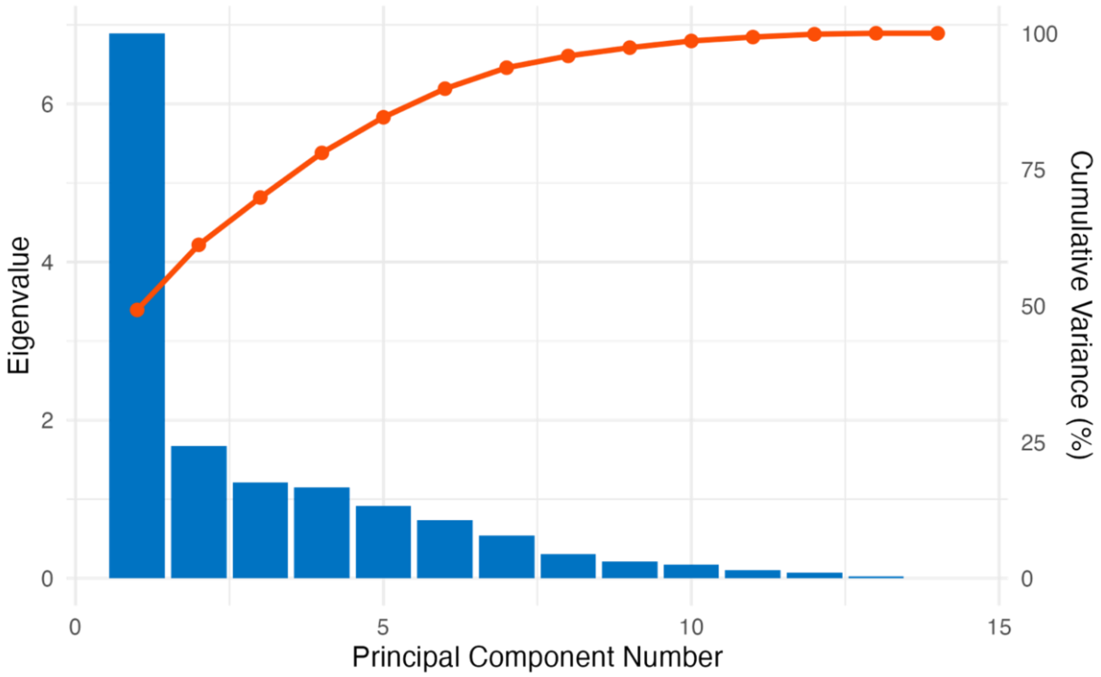

Fig. S1. Variance explained by principal components in the PTE dataset. PC1 accounts for nearly 50% of the total variance, highlighting its dominant role in capturing contamination patterns.

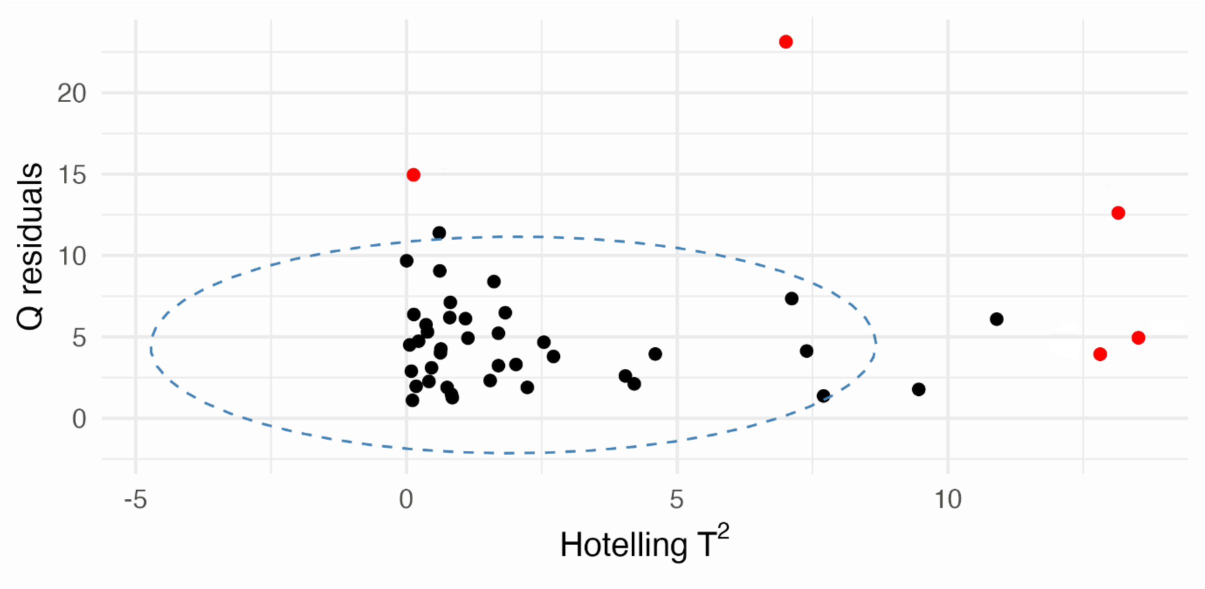

Fig. S2. Scatterplot of Hotelling's T^2 against Q-residuals for all 44 soil samples, using the first two principal components of the CLR-transformed PTEs concentrations. The dashed ellipse marks the 95 % confidence boundary (Mahalanobis distance), and the five samples falling outside that region are highlighted in red as outliers.

Table S2. Principal component (PC) loadings for the retained components (eigenvalue > 1) at each sampling site in the Scarlino Plain, showing the correlation (factor) loadings of each PTEs on each PC together with the percent variance explained by each component; and the corresponding enrichment factors (EFs) for each element, calculated relative to the local soil baseline concentrations.

Site	Principal Components				Enrichment Factors													
	1	2	3	4	As	Cd	Co	Cr	Cu	Mn	Hg	Ni	Pb	Sb	Sn	Tl	V	Zn
0	0.63	-0.73	0.66	-1.04	2.4	0.8	0.7	0.5	0.6	1.2	1.0	0.4	1.5	1.0	1.2	0.5	0.5	0.9
1	-2.59	1.09	0.53	0.34	4.9	1.9	1.3	0.6	2.0	0.5	1.0	1.1	5.5	1.0	1.5	1.2	1.0	3.3
2	-1.68	0.32	0.00	-0.43	5.7	1.8	1.1	0.6	1.8	1.1	1.4	0.9	2.3	1.0	1.5	1.1	1.0	3.3
3	0.86	-0.21	0.84	0.41	2.3	1.2	1.0	1.0	1.1	1.2	1.0	1.2	1.3	1.0	1.5	0.7	1.2	1.6
4	-0.79	0.10	-0.59	-0.93	3.7	1.4	0.8	0.5	1.1	1.0	1.0	0.7	1.4	1.0	1.0	0.8	0.6	1.7
5	1.70	0.09	1.43	0.02	2.2	0.6	0.9	1.0	1.2	0.9	1.0	1.1	1.1	1.0	1.3	0.5	1.2	1.3
6	-1.54	-0.41	0.92	1.64	2.0	1.9	1.5	0.8	2.0	1.3	1.0	1.2	3.5	1.0	1.9	0.9	1.2	3.5
7	-0.98	0.30	0.88	-0.92	5.9	1.7	1.5	1.1	2.3	1.7	1.2	1.6	2.4	1.2	1.2	0.6	1.9	2.8
8	-2.25	0.76	1.03	-0.92	5.5	1.6	1.1	0.7	1.9	1.3	1.0	1.1	4.8	1.0	1.2	0.5	1.1	2.2
9	-0.01	0.08	0.22	-2.06	3.2	0.7	0.8	0.4	0.6	0.8	1.0	0.5	2.2	1.0	0.8	0.5	0.6	1.2
10	-0.61	-0.64	-0.55	-0.46	4.1	1.4	1.3	0.6	1.4	1.5	1.1	1.1	2.0	1.0	1.3	1.2	1.0	1.9
11	-1.53	1.42	-1.96	0.69	1.5	1.8	0.8	0.8	1.7	1.0	1.0	0.9	2.8	1.0	1.0	1.4	0.8	2.6
12	0.64	0.15	-1.63	-0.26	1.6	1.4	1.1	1.0	0.9	1.4	1.0	1.0	1.9	1.0	1.0	1.3	1.0	1.3
13	2.11	-1.57	-0.30	1.15	1.0	0.9	1.3	0.8	1.5	1.7	1.0	1.3	0.9	1.0	1.2	1.0	1.0	0.9
14	1.12	-0.03	-0.84	0.90	1.0	1.2	1.0	1.0	1.3	1.1	1.0	0.9	1.4	1.0	1.0	1.1	1.0	1.1
15	3.21	-1.52	-1.13	1.17	0.5	0.6	0.9	0.6	0.9	1.5	1.0	1.0	0.6	1.0	1.0	1.2	0.8	0.7
16	3.11	-1.79	-0.44	2.17	0.4	1.2	1.2	0.9	1.1	1.9	1.0	1.2	0.9	1.0	1.2	1.0	1.0	0.5
17	0.14	2.15	-1.07	0.55	1.3	0.9	0.7	1.0	1.2	0.5	1.0	0.7	1.9	1.0	1.0	1.2	1.0	2.7
18	1.64	-1.78	0.30	0.75	1.1	1.1	1.3	0.9	1.2	2.1	1.0	1.1	1.0	1.0	1.2	0.7	1.0	1.1
19	1.50	-1.25	1.08	-0.21	2.3	0.9	1.3	0.9	1.1	1.4	1.0	1.0	1.1	1.0	1.2	0.6	1.2	1.0
20	2.45	-1.31	-0.50	1.72	0.4	0.9	1.0	0.6	1.3	1.3	1.0	1.0	0.7	1.0	0.9	0.7	0.9	1.0
21	-3.47	3.23	-1.29	2.67	1.5	5.2	0.9	2.4	3.4	0.6	1.2	1.1	5.9	1.0	1.8	1.6	1.0	6.6
22	2.10	0.17	-1.28	0.23	1.0	0.6	1.0	1.2	1.2	1.1	1.2	0.8	1.2	1.0	0.9	1.2	1.1	1.3
23	3.59	0.52	-0.60	-0.02	0.9	0.6	0.9	1.2	0.6	0.6	1.0	1.2	0.9	1.0	0.8	1.0	1.1	0.8
24	1.91	0.80	-2.26	-0.11	1.1	0.6	0.9	0.7	0.7	0.4	1.0	1.0	0.9	1.0	0.7	1.7	0.6	1.9
25	4.21	0.96	0.53	-0.88	1.0	0.6	0.7	1.5	0.3	0.4	1.0	0.8	1.0	1.0	0.8	0.5	0.9	0.4
26	-8.82	-4.20	-0.87	0.89	18.7	13.1	4.6	0.3	6.1	8.3	1.4	0.6	6.3	1.0	2.9	2.1	0.6	20.9
27	3.42	-0.71	2.10	0.66	1.5	0.6	1.5	1.3	0.8	0.5	1.0	1.7	1.1	1.0	1.3	0.6	1.2	0.8
28	3.46	-0.07	0.33	-0.62	1.0	0.6	1.0	1.3	0.5	0.8	1.0	1.1	1.0	1.0	0.8	0.6	1.0	0.7
29	-1.11	-0.46	0.49	0.48	2.9	2.1	1.4	0.7	1.7	1.1	1.1	1.1	1.7	1.0	1.3	0.8	1.1	3.0
30	2.22	0.62	0.35	-0.58	2.3	0.6	1.0	0.9	1.0	0.7	1.2	1.4	1.5	1.2	1.2	0.9	1.1	1.3
31	3.97	0.65	0.42	-0.18	1.0	0.6	0.7	1.1	0.5	0.3	1.0	1.0	0.7	1.0	0.8	0.6	0.8	0.5
32	-3.25	0.92	1.38	0.28	6.4	2.4	1.2	0.9	4.6	0.9	1.3	1.1	2.9	1.0	1.4	0.5	1.1	4.1
33	-1.68	3.03	2.92	1.27	6.2	2.3	1.2	0.6	1.8	0.0	1.0	0.9	2.3	1.0	1.3	0.7	1.0	3.6
34	-1.22	0.32	-0.08	-1.61	5.9	1.6	0.9	0.5	1.4	1.0	1.2	1.0	1.5	1.0	0.9	0.6	0.8	2.2
35	-4.64	-0.64	1.58	-0.14	9.2	3.8	1.6	0.5	2.2	1.2	1.1	0.8	3.6	1.0	1.8	0.6	0.8	7.8
36	-3.77	-0.19	0.09	0.44	5.3	3.0	1.9	0.7	2.2	1.4	1.0	1.1	5.9	1.0	1.7	1.2	1.1	5.8
37	-0.81	-0.07	-0.55	-1.80	11.4	1.5	1.4	0.7	1.7	1.2	1.4	1.2	1.9	1.2	1.2	1.3	1.1	2.1
38	-0.98	-0.01	-1.49	-1.61	7.0	1.1	1.1	0.5	1.3	1.3	1.1	1.0	1.8	1.0	1.0	1.5	0.8	2.1
39	0.43	-1.57	1.06	-0.50	4.5	1.2	1.3	0.7	1.3	1.5	1.0	1.2	1.1	1.0	1.3	0.6	0.9	1.1
40	-2.13	1.10	-1.90	-1.02	4.1	2.2	1.0	0.5	1.6	0.9	1.0	0.9	2.6	1.0	0.8	1.3	0.9	2.1
41	-2.67	0.30	-0.01	-0.54	7.4	3.0	1.5	0.8	2.1	1.2	1.2	1.3	3.6	1.0	1.3	1.0	1.1	3.1
42	1.61	1.38	0.37	0.46	1.3	0.7	0.7	0.7	1.5	0.4	1.0	0.8	1.1	1.0	0.8	0.6	1.2	1.0
43	0.50	-1.30	-0.17	-2.07	6.2	0.9	1.3	0.5	1.1	1.9	1.4	1.1	1.3	1.2	1.1	0.8	0.8	1.4

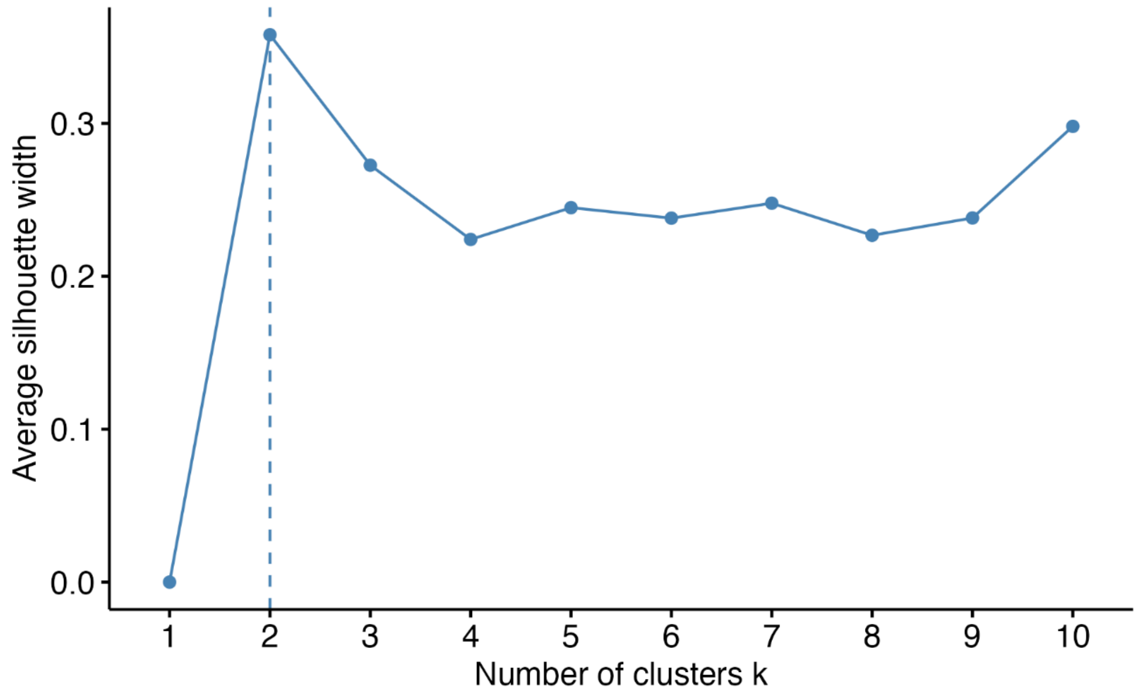

Fig S3. Average silhouette width as a function of the number of k-means clusters (k) applied to the centred log-ratio–transformed PTE concentration data. The dashed vertical line at k = 2 highlights the optimal clustering solution, corresponding to the highest mean silhouette width.

Table S3. Summary of the two k-means clusters derived from centered log-ratio PCA of potentially toxic element (PTE) concentrations in the Scarlino Plain soils. For each cluster, the table reports: (1) the symbol/colour used in Fig. 5b; (2) the mean PC1 and PC2 scores; (3) the dominant metals identified by significant z-clr loadings; (4) the average distance of sites in the cluster from the former Casone pyrite-roasting plant; and (5) the inferred source signature for each cluster.

Cluster	Symbol / colour on Fig. 5b	Mean PC 1	Mean PC 2	Dominant PTEs (z-clr)	Mean distance to former Casone plant (km)	Likely source signature
1	●	–3.43	+0.26	Pb ↑, Zn ↑, Cu ↑, Cd ↑, As ↑	1.4 km (closest)	Anthropogenic: fallout from historical pyrite roasting, traffic and other industrial activities
2	▲	+2.37	–0.41	Cr ↑, V ↑, Ni ↑, Mn–Co ↑	2.5 km (farthest)	Lithogenic / natural: soils developed with the influence of mafic–ultramafic substrates (ophiolites, basalts)

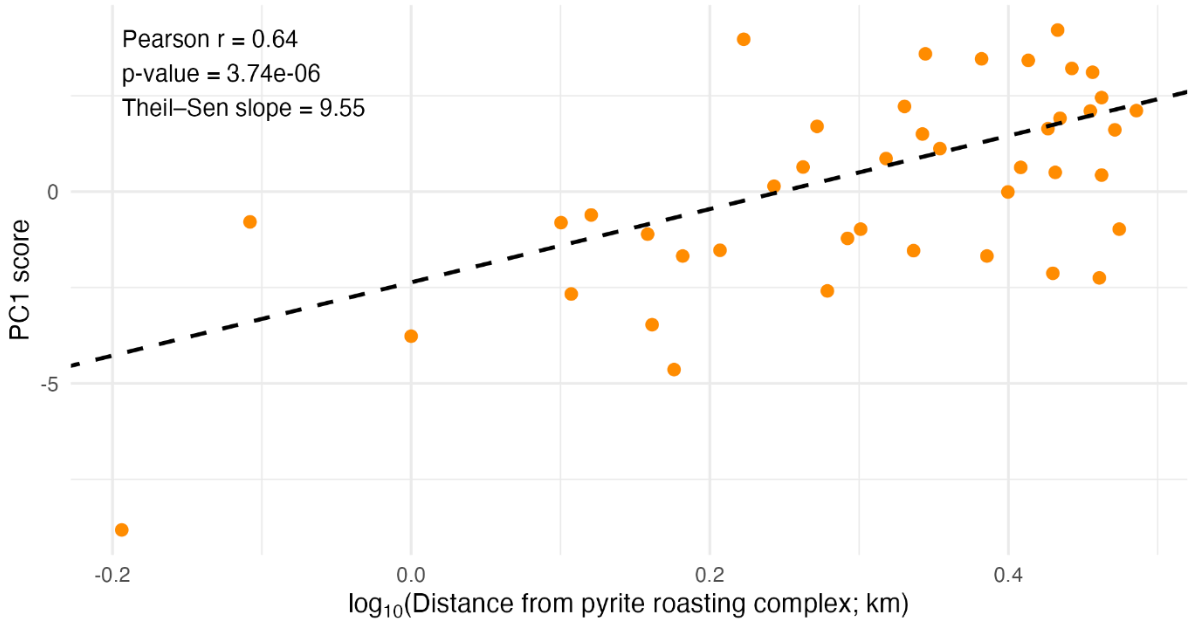

Fig. S4. Relationship between PC1 scores and $\log_{10}$-transformed distance from the former Casone pyrite-roasting complex. PC1, dominated by As, Cd, Cu, Pb, and Zn, decreases with smelter influence. Theil–Sen regression (dashed line) and Pearson's $r = 0.64$ ($p < 0.01$) indicate an exponential decline in PTEs enrichment with distance.
